# Livelihood opportunities amongst adults with and without disabilities in Cameroon and India: A case control study

**DOI:** 10.1371/journal.pone.0194105

**Published:** 2018-04-09

**Authors:** Islay Mactaggart, Lena Morgon Banks, Hannah Kuper, G. V. S. Murthy, Jayanthi Sagar, Joseph Oye, Sarah Polack

**Affiliations:** 1 International Centre for Evidence in Disability, London School of Hygiene & Tropical Medicine, London United Kingdom; 2 South Asia Centre for Disability Inclusive Development and Research, Indian Institute of Public Health Hyderabad, Hyderabad, Telangana, India; 3 Sightsavers Cameroon, Yaounde, Cameroon; University of Illinois at Urbana-Champaign, UNITED STATES

## Abstract

Proven links between disability and poverty suggest that development programmes and policies that are not disability-inclusive will leave persons with disabilities behind. Despite this, there is limited quantitative evidence on livelihood opportunities amongst adults with disabilities in Low and Middle Income Countries. This study adds to the limited evidence base, contributing data from one African and one Asian Setting. We undertook a population-based case–control study of adults (18+) with and without disabilities in North-West Cameroon and in Telangana State, India. We found that adults with disabilities were five times less likely to be working compared to age-sex matched controls in both settings. Amongst adults with disabilities, current age, marital status and disability type were key predictors of working. Inclusive programmes are therefore needed to provide adequate opportunities to participate in livelihood prospects for adults with disabilities in Cameroon and India, on an equal basis as others. These findings are of crucial importance at this stage of the Sustainable Development Agenda, to ensure that the mandate of inclusive development is achieved.

## Introduction

The 2030 Sustainable Development Agenda (SDA) adopted by the United Nations General Assembly in September 2015 asserts that it shall leave no-one behind in its global push for social and economic development[1]. As part of the agenda for ending poverty and inequality, ‘decent work for all’ has been promoted in Sustainable Development Goal (SDG) Eight as a key tool for inclusive economic development[1]. This rhetoric is of crucial importance in relation to the estimated one billion people living with disabilities globally, 80% of whom live in low and middle income countries (LMICs) [2, 3].

Persons with disabilities are described in the United Nations Convention on the Rights of Persons with Disabilities (UNCRPD) as those who have ‘long-term physical, mental, intellectual or sensory impairments which in interaction with various barriers may hinder their full and effective participation in society on an equal basis with others’ [4]. Disability was largely absent from the international development agenda set by the precedent 2000–2015 Millennium Development Goals, which arguably led to persons with disabilities being excluded from development efforts and widening the poverty gap between persons with and without disabilities [5–7]. In response to criticism on this issue, the 2015–2030 SDGs have placed a greater emphasis on inclusive development, with calls for tracking equity in progress towards each goal through disaggregation of data by disability.

Poverty and disability are interlinked; a recent systematic review showed a positive association between disability and economic poverty in 80% of the 78 included studies[8]. The relationship between poverty and disability is theorized as cyclical [9, 10]. Poverty is posited to increase the risk of disability via exclusion from health care or health information, and through heightened exposure to risk factors for poor health and impairment (including trauma, infectious disease, unsafe environments, poor sanitation and malnutrition) [11, 12]. Conversely, disability can lead to or exacerbate poverty through participation restrictions, including exclusion from education, and barriers to engaging in decent work [13]. Exclusion from livelihood opportunities has also been shown to negatively impact on psychosocial wellbeing, identity and social inclusion [14].

Livelihoods can be defined as the means through which individuals or households are able to meet their basic needs. It encompasses not only remunerated labour, but also an individual’s capabilities (e.g. level of education, skills), assets and participation in other productive activities (e.g. farming for direct consumption)[15]. Building upon this definition, the Sustainable Livelihood Approach promotes the idea that for a livelihood to be sustainable, individuals must be able to both maintain a basic standard of living through times of stress and shock (e.g. natural disasters, economic upheaval) and to have opportunities for livelihood improvement (e.g. through education and productive investments). The Sustainable Livelihood Approach has been a fundamental cornerstone of international development and poverty reduction, as it emphasizes a shift beyond the subsistence level, toward long-term poverty alleviation. A key component for sustainable livelihoods is engagement in decent work: work that is stable, respects an individual’s dignity, provides safe conditions and has fair remuneration.

Persons with disabilities are believed to face widespread exclusion from livelihood opportunities in many settings. While there is clear evidence from high-income countries of a gap in the employment rate between persons with and without disabilities–averaging 40% of the rate of persons without disabilities–equivalent analyses from LMICs are more challenging given the complexity of livelihood situations in many of these settings [16–18]. Notably, in many LMICs the vast majority of the labour force participates in the informal economy, in subsistence agriculture, or in economic activities that are not well monitored and difficult to measure [19]. Still, existing evidence points to substantial inequalities: for example, in the 2002–2003 World Health Surveys significant employment gaps between persons with and without disabilities were found across nine of 15 LMICs, with persons with multiple impairments and men experiencing the highest gaps[20]. Lower rates of employment among persons with disabilities have been found consistently in other studies, although many focus on formal sector employment only[21, 22].

Considering the over-representation of persons with disabilities amongst the poor[23] and the SDA’s focus on decent work for all as a tool for inclusive economic development, there is an urgent need for data on access to livelihood opportunities amongst persons with disabilities. Understanding this relationship is key if the focus on inclusive development and elimination of poverty within the SDGs is to be achieved.

Cameroon and India are two countries classified by the World Bank as lower middle income, on account of gross national income per capita, with approximately a quarter of the population in both countries living below $1.25 per day [24]. The United Nations Human Development Index—which generates country-level composite scores using indicators of health, life expectancy, education, and standard of living—ranked India 131st and Cameroon 153rd in 2016[24].

The UNCRPD was signed by Cameroon in 2008, but is yet to be ratified[25]. Limited data on disability in Cameroon are available. However, a recent review by Ray et al. (2017) identified five studies that addressed access to work amongst persons with specific impairments (for example visual impairments or related to HIV) in Cameroon, all of which documented unequal employment opportunities related to a combination of stigma, participation restrictions and restricted access to education[26].

The Persons with Disabilities Act in India, which legislates the right to equal opportunities and full participation amongst persons with disabilities, was enforced by the National Government of India in 1995[27]. This was followed by a National Policy for Persons with Disabilities in 2006 and ratification of the UNCRPD in 2007[27] [25]. However, ineffective programmes, insufficient funding and complexities in resource mobilisation are all stated barriers to the realisation of rights as set out in Indian inclusive legislature, leading to lower employment participation rates amongst persons with disabilities in the country [28–30].

The aim of this study was to build evidence on access to livelihoods amongst adults with and without disabilities in one district each of two LMICs: Cameroon and India.

## Methods

### Study overview

This was an observational population-based study in two LMIC settings. We undertook all-age population-based surveys of disability in a district each of one African and one Asian LMIC (Cameroon and India respectively). Disability was conceptualised as per the World Health Organisation’s (WHO) bio-psycho-social International Classification of Functioning, Disability and Health (ICF), which perceives disability as an umbrella term incorporating difficulties in any one of three inter-related spheres of functioning–impairments, activity limitations or participation restrictions–as the result of an interaction between a health condition and contextual factors[31]. We used both a self-reported functioning tool and clinical screening tools to identify individuals with disabilities.

A case-control study of adults with and without disabilities (cases and controls, respectively) was nested within the population-based study. Participants responded to questions concerning their current livelihood situation, so as to assess the impact of disability on livelihood opportunities and wellbeing.

### Study setting

The study took place in Fundong Health District, North West Cameroon in 2013, and in Mahbubnagar District, Telangana State in India in 2014.

### Survey population and sampling

We conservatively estimated the all-age prevalence of disability to be four percent in both India and Cameroon[2, 32]. This required a sample of 4,056 per country, assuming precision of 20%, 95% confidence, a design effect of 1.4 and 20% non-response.

In each setting, we selected 51 primary sampling units (clusters) from the most recent National Census using probability proportionate to size sampling. Within clusters, modified compact segment sampling was used[33]. A cluster sketch map was created by enumerators, together with local leaders, and divided into segments of approximately 80 people. One segment was randomly selected, and all households within this segment were visited door-to-door until 80 people of all ages were enumerated.

Eligible household members were informed about the survey and invited to attend a local, central location for screening over the following two days. Enumerators made two repeat visits as needed to encourage attendance and those physically unable to attend (e.g. due to mobility impairment) were visited by the survey team in their homes at the end of the second day.

### Screening for disability

Participants were screened for i) self-reported functional limitations and ii) clinical impairments in vision, hearing and the musculoskeletal system, epilepsy and depression. Epilepsy as a health condition was included due to the documented associations both between epilepsy and health-related quality of life, and between seizure-related falls and long-term physical impairment [34]. The screening tools and protocols for adults (18 and above) are described below [35].

#### Self-reported limitations

The Washington Group Extended Set on Functioning for Adults was used to screen for self-reported functional limitations. This is comprised of 21 questions about level of difficulty with different domains of functioning (e.g. seeing, hearing), and scored on a severity scale of no difficulty, some difficulty, a lot of difficulty and cannot do [36].

#### Vision impairment

We assessed visual acuity (VA) using a tumbling ‘E’ chart with size 6/18 optotype on one side and 6/60 on the other[37]. Visual impairment was categorised using the WHO protocol for VA in the better eye: moderate VA <6/18; severe VA <6/60 and >3/60; blind VA <3/60.

#### Hearing impairment

Hearing impairment was measured using a modified version of the WHO Ear and Hearing Disorders Survey Protocol[38]. All participants were first tested using an otoacoustic emissions (OAE) machine for middle ear function. All participants who failed this test in both ears, or for whom an OAE reading could not be taken, underwent Pure Tone Audiometry testing using a field audiometer. Hearing thresholds were recorded as the average threshold across four test frequencies (1KHz, 2KHz, 4KHz and 0.5KHz) and categorised as per WHO recommendations for hearing thresholds in A-weighted decibels (dBA) for the better ear: moderate 41–60 dBA; severe 61–80 dBA; profound >80 dBA.

#### Musculoskeletal impairment and epilepsy

Musculoskeletal impairment and epilepsy were both assessed using the Rapid Assessment of Musculoskeletal Impairment protocol[39].This comprises six preliminary screening questions on a) difficulty using the musculoskeletal system b) use of a mobility aid c) whether a body part was considered misshapen by the participant and d) past experience of seizures. In India, an additional screening question on chronic back pain was added. Any participant responding affirmatively to one or more question was examined by an Orthopaedic Clinical Officer (Cameroon) or physiotherapist (India), including standardised observation of activities and history to determine the presence of moderate or severe physical impairment and/or epilepsy.

#### Clinical depression

Clinical depression was measured using the Patient Health Questionnaire (PHQ-9), previously validated in both settings[40]. All participants answered three initial screening questions, with a further six questions triggered based on affirmative response. A composite score of 20 or above signifies symptoms of severe depression.

#### Defining disability

For the purposes of this study, participants were considered to have a disability if they met any of the following criteria:

- Self-reported functional limitations: ‘A lot of difficulty’ or ‘cannot do’ in any basic activity domain (seeing, hearing, walking or climbing steps, understanding, being understood, remembering, concentrating, self-care, upper body strength and fine motor dexterity).
- Vision Impairment: presenting vision in better eye of <6/18
- Hearing Impairment: Presenting average hearing threshold in better ear of >40dBA
- Musculoskeletal Impairment: structure impairment with moderate effect on the musculoskeletal system’s ability to function as a whole 25–49% or greater
- Epilepsy: three or more tonic clonic seizures previously experienced
- Depression: score of 20 or above on PHQ-9

### Nested case-control study

All participants aged 18 and above who screened positive for disability were invited to participate in the nested case-control study (“cases”) alongside an age (+/- five years), sex and cluster-matched “control” without a disability. One additional adult with a disability was identified through community key informants (e.g. local health workers) from an adjacent segment in each cluster to ensure adequate sample size for the case-control study.

Cases and controls were interviewed using a standardised questionnaire including modules on: socio-economic status, education, healthcare and rehabilitation, participation and environmental barriers, water and sanitation as well as livelihoods, which is the focus of this paper (S1 File and S2 File). The livelihoods module assessed engagement in work in the last 12 months, type of work (including informal work seasonality and type of payment), reasons if not working and access to both state and non-state livelihood support. Questionnaires were translated into local languages using standard forward and backward translation procedures and were pilot tested in each setting.

### Training

Three teams per setting received ten days training. Teams were comprised of two interviewers, two enumerators, three field assistants, one audiologist/ENT nurse, one ophthalmic nurse/vision tech and one physiotherapist/orthopaedic clinical officer

### Data entry and analysis

Cases included all adults (18+) with disabilities identified in the population-based survey component of the study, plus one additional adult with a disability per cluster identified via case-finding. One age (+/- 5 years), sex and community matched control was also recruited into the study per case. All screening data were double-entered into a purpose-built Microsoft Access Database. The case-control questionnaire was built using Open Data Kit software and administered using ASUS Google Nexus 7 android tablets. Data were analysed in STATA 12.0.

The primary outcome variable ‘working’ was defined as having undertaken any activities contributing to household consumption (inclusive of subsistence farming and remuneration for any activity in cash or kind).

Six binary, non-mutually-exclusive, variables for ‘type’ of disability were constructed based on a combination of the clinical and self-reported results. These were:

- Vision: VA<6/18, or reported ‘a lot of difficulty’ or ‘cannot do’ in the vision domain of the WG questions
- Hearing: Presenting average hearing threshold in better ear of >40dBA, or reported ‘a lot of difficulty’ or ‘cannot do’ in the hearing domain of the WG questions
- Physical Function: Structure impairment of 25–49% or greater, screens positive for epilepsy, or reported ‘a lot of difficulty’ or ‘cannot do’ in the physical domain of the WG questions
- Intellectual Function: Reported ‘a lot of difficulty’ or ‘cannot do’ in the learning and understanding domains of WG questions
- Depression: score of 20 or above on PHQ-9
- Multiple: More than one of the above.

Severity of limitation was calculated amongst cases as ‘moderate’ or ‘severe/profound’ based on severity combined across both the participant’s reported functional limitation responses (with ‘a lot of difficulty’ corresponding to moderate, ‘cannot do’ as severe) and clinical impairment severity as per the international protocols described above.

We constructed a socio-economic status (SES) score through principal component analysis (PCA) of household assets [41]. The PCA score distribution amongst controls was used to define the interquartile range, with cases then categorised into quartiles based on control ‘cut-points’[42].

We undertook logistic regression analyses adjusted for age and sex to a) compare participation in work between cases and controls stratified by age, sex, SES, marital status and education and b) to explore socio-demographic and clinical predictors of working amongst cases. We also undertook multivariate logistic regression analyses for the above relationships, incorporating all above variables in the model to adjust for potential confounders. Binary variables were created for marital status (married versus never married, widowed or divorced) and education (no education versus at least one year of education). Conditional logistic regression was not conducted since complete matching was not achieved. The ‘vce’ command was used to calculate robust standard errors accounting for the heteroscedasticity of the sample in relation to clustering.

### Ethical considerations

Ethical Approval for the study was provided by:

- The London School of Hygiene and Tropical Medicine (London, UK)
- National Ethics Committee for Research in Human Health (CNERSH, Cameroon)
- Cameroon Baptist Convention Health Board Institutional Review Board (Cameroon)
- Indian Institute of Public Health Hyderabad Institutional Ethics Committee (India)
- Government of India Health Ministry Screening Committee (India)

Informed written/finger-print consent was obtained from all participants. Participants identified in the screening to have vision, hearing or musculoskeletal impairments were examined by the relevant clinical team members to determine cause and referral needs. Clinical team members also distributed basic medicines where appropriate and all participants with unmet health or rehabilitative needs were referred to relevant services.

## Results

### Study population

In India, the sample comprised 441 adult cases (378 identified via the survey and 63 through case-finding) and 288 age and sex matched controls. In Cameroon, 315 adult cases (271 identified via the survey and 44 via case-finding) and 184 controls were identified. The total number of controls is lower than the number of cases in both settings due to high prevalence of disability in older adults, limiting the availability of eligible cluster-matched controls in this age group.

Cases were well matched to controls on sex in both settings, but were more likely to be in the oldest age category (66+) in both India (OR = 5.3, 95%CI = 3.2–8.9) and Cameroon (2.9, 1.9–4.4) (Table 1). Low levels of education and literacy were observed in both sites, with no differences between cases and controls.

**Table 1:** Socio-demographic characteristics of cases and controls in India and Cameroon.

	India			Cameroon		
	Cases (n = 441) N (%)	Controls (n = 288) N (%)	Age & Sex Adj OR (95% CI)	Cases (n = 315) N (%)	Controls (n = 184) N (%)	Age & Sex Adj OR (95% CI)
**Age Group**						
18–33	83 (19%)	76 (26%)	Baseline	54 (17%)	45 (25%)	Baseline
34–49	94 (21%)	84 (29%)	1.0 (0.8–1.4)	33 (10%)	42 (23%)	0.7 (0.4–1.0)
50–65	165 (37%)	111 (39%)	1.4 (1.1–1.7)	70 (22%)	51 (28%)	1.1 (0.8–1.7)
>65	99 (22%)	17 (6%)	5.3 (3.2–8.9)	158 (50%)	46 (25%)	2.9 (1.9–4.4)
**Sex**						
Male	199 (45%)	133 (46%)	Baseline	123 (39%)	70 (38%)	Baseline
Female	242 (55%)	155 (54%)	1.0 (0.9–1.2)	192 (61%)	114 (62%)	1.1 (0.8–1.6)
**Education**						
None	322 (73%)	186 (65%)	1.6 (1.0–2.7)	195 (62%)	78 (42%)	2.0 (1.0–3.9)
Primary	61 (14%)	37 (13%)	1.7 (1.0–2.9)	97 (31%)	77 (42%)	1.5 (0.8–2.6)
Secondary or higher	58 (13%)	65 (23%)	Baseline	23 (7%)	29 (16%)	Baseline
**Literacy**^**a**^						
Can read	124 (28%)	102 (36%)	0.8 (0.6–1.2)	113 (36%)	101 (55%)	0.7 (0.5–1.0)
Cannot read	317 (72%)	184 (64%)	Baseline	199 (64%)	82 (44.8%)	Baseline
**Marital status**^**a**^						
Married/ living together	327 (74%)	239 (84%)	Baseline	170 (54%)	116 (63%)	Baseline
Divorced/ Separated	8 (2%)	7 (2%)	0.7 (0.3–1.7)	7 (2%)	7 (4%)	0.7 (0.2–2.4)
Widowed	60 (14%)	17 (6%)	1.6 (0.9–2.6)	73 (23%)	31 (17%)	1.2 (0.7–2.1)
Never married	46 (10%)	23 (8%)	2.6 (1.3–5.3)	62 (20%)	29 (16%)	3.6 (1.6–8.3)
**SES**						
1^st^ Quartile (poorest)	155 (36%)	72 (25%)	1.6 (1.1–2.4)	78 (25%)	46 (25%)	1.0 (0.5–2.0)
2^nd^ Quartile	81 (19%)	72 (25%)	0.8 (0.5–1.3)	113 (36%)	46 (25%)	1.6 (0.8–3.3)
3^rd^ Quartile	103 (24%)	72 (25%)	1.1 (0.6–1.8)	62 (20%)	46 (25%)	0.8 (0.4–1.7)
4^th^ Quartile (richest)	95 (22%)	72 (25%)	Baseline	62 (20%)	46 (25%)	Baseline
**Disability measure** ^b^						
Vision	170 (39%)	-	-	108 (34%)	-	-
Hearing	175 (40%)	-	-	120 (38%)	-	-
Physical Function	243 (55%)	-	-	190 (60%)	-	-
Intellectual Function	67 (15%)	-	-	60 (19%)	-	-
Depression	41 (9%)	-	-	8 (3%)	-	-
Multiple	174 (39%)	-	-	115 (36%)	-	-
**Disability onset**						
Under 5	172 (41%)	-	-	47 (15%)	-	-
Childhood (5–17)	36 (9%)	-	-	23 (7%)	-	-
Working age (18–49)	78 (19%)	-	-	73 (23%)	-	-
Older age (50 +)	103 (25%)	-	-	125 (40%)	-	-
Unknown	29 (7%)	-	-	43 (14%)	-	-
**Functional limitation Severity**^**c**^						
Moderate	223 (56%)	-	-	238 (76%)	-	-
Severe/Profound	182 (44%)	-	-	74 (24%)	-	-

^a^Missing marital status and literacy status for two controls in India, and for three cases and one control in Cameroon

^b^ Not mutually exclusive (i.e. sum >100%)

^c^India: 26 severity missing as Epilepsy only cases with no severity scale, 3 missing Cameroon

The principal component on which the socio-economic status (SES) index was derived comprised an eigenvalue of 5.99 and explained 21% of the variance, supporting its suitability in representing SES (data not shown).

Cases in India were more likely to be in the poorest socio-economic quartile than controls (1.6, 1.1–2.4) but there were no differences in Cameroon. In both settings persons with disabilities were much more likely to have never married (India: 2.6 (1.3–5.3), Cameroon: 3.6 (1.6–8.3)).

Among persons with disabilities, the distribution of ‘type’ of disability experienced was similar in both countries. Physical limitations accounted for the highest proportion of disability in both samples (55% of cases in India and 60% in Cameroon), followed by sensory limitations (vision 39%, hearing 40% in India, vision 34%, hearing 38% in Cameroon), intellectual limitations (15% and 19% respectively) and depression (9%, 3%). One third of persons with disabilities experienced multiple limitations. Due to case-finding, these do not constitute prevalence estimates or population-reliable proportions. Prevalence estimates from the population-based survey are reported elsewhere[43].

Amongst persons with disabilities, reported age of onset was lower in India than Cameroon (41% within the first five years of life in India, compared with 15% in Cameroon). Mean years of disability experienced was therefore higher in India (27.6, standard deviation 24.6) than Cameroon (17.7, sd 18.9). In India, 56% of persons with disabilities experienced moderate functional limitations compared with 76% in Cameroon, with the remainder in each setting experiencing severe or profound functional limitation.

### Livelihoods

Persons with disabilities were substantially less likely to have engaged in work (including informal activities or subsistence agriculture) in the past 12 months compared to persons without in both India (82% of controls versus 48% of cases, OR = 0.2, 95% CI = 0.2–0.4) and Cameroon (90% versus 69%, 0.3, 02–0.5) (Table 2). This relationship remained when stratified by age group, sex, marital status and education level. Persons with disabilities were significantly less likely than persons without to work across the SES quartiles with the exception of the second-lowest (poorest) socio-economic quartile in India (0.5, 0.2–1.2) and highest (least poor) quartile in Cameroon (0.7, 0.3–1.7) where the differences were non-significant.

**Table 2:** Relationship between disability and working status stratified by age, sex, education and SES (% worked in the last twelve months).

	India			Cameroon		
	Cases (n = 441) N (%)	Controls (n = 288) N (%)	Age & Sex Adj OR (95% CI)	Cases (n = 315) N (%)	Controls (n = 184) N (%)	Age & Sex Adj OR (95% CI)
**Total study sample**	212 (48%)	235 (82%)	0.2 (0.2–0.4)	217 (69%)	165 (90%)	0.3 (0.2–0.5)
**Sex**						
Male	111 (56%)	118 (89%)	0.2 (0.1–0.4)	87 (71%)	65 (93%)	0.2 (0.1–0.6)
Female	101 (42%)	117 (75%)	0.3 (0.2–0.5)	130 (68%)	100 (88%)	0.3 (0.2–0.6)
**Age (years)**						
18–33	40 (48%)	56 (74%)	0.3 (0.1–0.7)	29 (54%)	40 (89%)	0.1 (0.1–0.4)
34–49	79 (84%)	79 (94%)	0.3 (0.1–1.1)	29 (88%)	38 (90%)	0.7 (0.2–3.3)
50–65	79 (48%)	91 (82%)	0.2 (0.1–0.4)	56 (80%)	48 (94%)	0.2 (0.1–0.8)
>65	14 (14%)	9 (53%)	0.1 (0.1–0.4)	103 (65%)	39 (85%)	0.3 (0.1–0.8)
**Marital Status**						
Married	180 (55%)	207 (87%)	0.4 (0.2–0.9)	129 (76%)	109 (94%)	0.3 (0.1–0.7)
Not Married	32 (28%)	28 (57%)	0.2 (0.1–0.3)	88 (61%)	56 (82%)	0.3 (0.2–0.6)
**Education**						
One or more years education	65 (55%)	80 (78%)	0.2 (0.1–0.4)	89 (74%)	95 (90%)	0.4 (0.2–0.8)
No education	147 (46%)	115 (83%)	0.3 (0.1–0.6)	128 (66%)	70 (90%)	0.2 (0.09–0.6)
**SES**						
1^st^ Quartile (poorest)	79 (51%)	63 (88%)	0.1 (0.05–0.4)	50 (64%)	41 (89%)	0.2 (0.08–0.6)
2^nd^ Quartile	45 (56%)	56 (78%)	0.5 (0.2–1.2)	79 (70%)	44 (96%)	0.1 (0.02–0.5)
3^rd^ Quartile	53 (52%)	64 (89%)	0.1 (0.05–0.3)	44 (71%)	43 (93%)	0.2 (0.06–0.6)
4^th^ Quartile (richest)	30 (32%)	52 (72%)	0.2 (0.07–0.4)	44 (71%)	37 (80%)	0.7 (0.3–1.7)

Amongst study participants that were working, there was no difference in the type of work undertaken (work for self/household business, work for non-household member or work on farm owned/rented by the household) by persons with and without disabilities in either setting. In India, persons with disabilities were more likely (2.0, 1.3–3.1) to work irregularly (i.e. seasonally/part of the year rather than throughout), and less likely to be paid in a combination of cash funds and in kind than persons without disabilities (Table 3, next page). Amongst those working, half (50%) of both persons with and without disabilities worked on a farm either owned or rented by the household, compared with over three quarters of both persons with and without disabilities in Cameroon.

**Table 3:** Relationship between disability and livelihoods.

	India^a^			Cameroon^a^		
	Cases (n = 212)	Controls (n = 233)	Age & Sex Adj OR (95% CI)	Cases (n = 214)	Controls (n = 163)	Age & Sex Adj OR (95% CI)
	N (%)	N (%)		N (%)	N (%)	
**Type of work**^**b**^						
Work for self/ household business	18 (8%)	28 (12%)	Baseline	31 (14%)	30 (18%)	Baseline
Work for non household member	88 (42%)	88 (38%)	1.6 (0.8–3.2)	18 (8%)	11 (7%)	1.6 (0.7–3.6)
Work on farm owned or rented by household	106 (50%)	117 (50%)	1.4 (0.7–2.9)	165 (77%)	122 (75%)	0.7 (0.4–1.5)
**Regularity of work**						
Throughout the year	117 (55%)	165 (71%)	Baseline	95 (44%)	84 (52%)	Baseline
Seasonally/ part of the year	88 (42%)	62 (27%)	2.0 (1.3–3.1)	99 (46%)	66 (40%)	1.1 (0.7–1.9)
Once in a while	7 (3%)	6 (3%)	1.7 (0.6–4.9)	20 (9%)	13 (8%)	1.4 (0.6–3.3)
**Type of payment**^**b**^						
Cash onlyCash only	166 (78%)	153 (66%)	Baseline	25 (12%)	21 (13%)	Baseline
Cash and in kind	40 (19%)	71 (30%)	0.5 (0.3–0.8)	87 (41%)	65 (40%)	0.8 (0.4–1.6)
In kind only	4 (2%)	7 (3%)	0.5 (0.1–1.6)	42 (20%)	31 (19%	0.8 (0.3–1.7)
Not paid	2 (1%)	2 (1%)	-	60 (28%)	46 (28%)	0.7 (0.3–1.6)

‘-’Omitted due to small cell size

^a^missing data on livelihoods for two controls in India, and three cases and two controls in Cameroon, excluded from analysis

^b^Amongst all those working within last 12 months

Table 4 explores potential predictors of working amongst persons with disabilities. In India, persons with disabilities aged 34–49 (36.3, 15.7–83.6) or 50–65 (5.8, 3.0–11.0) compared to over 65 years, and those who were married compared to those who weren’t (2.3, 1.4–4.0), were more likely to work. Females (0.5, 0.3–0.7) and persons in the highest socio-economic quartile were significantly less likely to work, while there was no significant association with education. In terms of disability, those who reported onset of disability aged fifty and above (0.3, 0.1–0.6) and those experiencing physical limitations (0.4, 0.2–0.6) or depression (0.3, 0.1–0.7) were the least likely to be working. Similarly, in Cameroon, the likelihood of working amongst persons with disabilities was highest in the age groups of 34–49 (3.9, 1.4–11.1) and 50–65 (2.2, 1.1–3.9), and amongst those who were married (2.0, 1.1–3.6). There was no relationship between likelihood of working and sex or socio-economic status in Cameroon, but education was positively associated with working (2.0, 1.1–3.6). Persons with disabilities in Cameroon were more likely to be working if they acquired their disability in later childhood (aged 5–17) compared to under the age of five (3.6, 1.2–10.8) and were less likely to be working if they experienced physical limitations (0.4, 0.2–0.6) or multiple limitations (0.4, 0.2–0.7). Persons with severe or profound functional limitations were also less likely to work compared to those with moderate functional limitations (0.4, 0.2–0.8). These results remained similar with multivariate adjustment (data not shown).

**Table 4:** Predictors of working in the last twelve months among cases.

	India			Cameroon		
	Working (n = 212) N (%)	Not working (n = 229) N (%)	Age & Sex Adj OR (95% CI)	Working (n = 217) N (%)	Not working (n = 98) N (%)	Age & Sex Adj OR (95% CI)
**Sex**						
Male	111 (52%)	88 (38%)	Baseline	87 (40%)	36 (37%)	Baseline
Female	101 (48%)	141 (62%)	0.5 (0.3–0.7)	130 (60%)	62 (63%)	0.9 (0.5–1.5)
**Age (years)**						
18–33	40 (19%)	43 (19%)	5.6 (2.6–11.9)	29 (13%)	25 (26%)	0.6 (0.3–1.2)
34–49	79 (37%)	15 (7%)	36.3 (15.7–83.6)	29 (13%)	4 (4%)	3.9 (1.4–11.1)
50–65	79 (37%)	86 (38%)	5.8 (3.0–11.0)	56 (26%)	14 (14%)	2.2 (1.2–3.9)
>65	14 (7%)	85 (37%)	Baseline	103 (47%)	55 (56%)	Baseline
**Marital Status**						
Married	180 (85%)	147 (64%)	2.3 (1.4–4.0)	129 (59%)	41 (42%)	2.0 (1.1–3.6)
Not Married	32 (15%)	82 (36%)	Baseline	88 (41%)	57 (58%)	Baseline
**Education**						
Educated	65 (31%)	54 (24%)	0.9 (0.4–1.7)	89 (41%)	31 (32%)	2.0 (0.9–4.2)
Not educated	147 (69%)	175 (76%)	Baseline	128 (59%)	67 (68%)	Baseline
**SES**						
1^st^ Quartile (poorest)	79 (33%)	79 (38%)	Baseline	50 (23%)	28 (29%)	Baseline
2^nd^ Quartile	36 (16%)	45 (22%)	1.4 (0.7–3.1)	79 (36%)	34 (35%)	1.4 (0.8–2.4)
3^rd^ Quartile	50 (50%)	53 (26%)	1.0 (0.6–1.7)	44 (20%)	18 (18%)	1.4 (0.7–2.8)
4^th^ Quartile (richest)	65 (29%)	30 (15%)	0.4 (0.2–0.8)	44 (20%)	18 (18%)	1.3 (0.7–2.3)
**Age of Disability onset**						
Under 5	98 (53%)	74 (37%)	Baseline	26 (14%)	21 (26%)	Baseline
Childhood (5–17)	12 (6%)	24 (12%)	0.5 (0.2–1.6)	19 (10%)	4 (5%)	3.6 (1.2–10.8)
Working age (18–49)	54 (29%)	24 (12%)	1.1 (0.6–2.0)	54 (29%)	19 (24%)	1.4 (0.6–3.6)
Older age (50 +)	22 (12%)	81 (40%)	0.3 (0.1–0.6)	89 (47%)	36 (45%)	1.6 (0.7–3.9)
**Disability Type**^**a**^						
Vision	77 (36%)	93 (41%)	1.3 (0.8–2.1)	76 (35%)	32 (33%)	1.1 (0.6–2.0)
Hearing	84 (40%)	91 (40%)	1.5 (1.0–2.4)	79 (36%)	41 (42%)	0.9 (0.6–1.5)
Physical Function	94 (44%)	149 (65%)	0.4 (0.2–0.6)	117 (54%)	73 (74%)	0.4 (0.2–0.6)
Intellectual Function	34 (16%)	33 (14%)	0.9 (0.5–1.7)	35 (16%)	25 (26%)	0.6 (0.3–1.0)
Depression	10 (5%)	31 (14%)	0.3 (0.1–0.8)	6 (3%)	2 (2%)	-
Multiple	62 (29%)	112 (49%)	0.6 (0.4–1.0)	65 (30%)	50 (51%)	0.4 (0.2–0.7)
**Functional Limitation Severity**^**b**^						
Moderate	117 (61%)	116 (52%)	Ref.	174 (81%)	64 (65%)	Ref.
Severe/Profound	75 (39%)	107 (48%)	0.8 (0.5–1.2)	40 (19%)	34 (35%)	0.4 (0.2–0.8)

^a^Non mutually exclusive binary variables

^b^Three missing severity Cameroon; 26 missing severity India excluded from this analysis

There were differences between persons with and without disabilities in the reasons for not working (p<0.001, Table 5). In both settings, persons with disabilities not working commonly reported ageing (India: 44%, Cameroon: 22%) and their health or disability (India: 35%, Cameroon: 60%) as the primary reason. Persons without disabilities more frequently reported not working due to undertaking unpaid activities (such as housework) (India: 47%, Cameroon: 37%) and ageing (India: 38%, Cameroon:26%).

**Table 5:** Primary reason not working amongst those who have not worked at all in the past 12 months.

	India				Cameroon			
	All (n = 282) N (%)	Cases (n = 229) N (%)	Controls (n = 53) N (%)	p-value	All (n = 117) N (%)	Cases (n = 98) N (%)	Controls (n = 19) N (%)	p-value^a^
**Unpaid activities**^**b**^	42 (12%)	17 (7%)	25 (47%)	<0.001	14 (12%)	7 (7%)	7 (37%)	<0.001
**Ageing/retirement**	121 (43%)	101 (44%)	20 (38%)		27 (23%)	22 (22%)	5 (26%)	
**Health or disability**	85 (30%)	80 (35%)	5 (9%)		64 (55%)	59 (60%)	5 (26%)	
**Other**	34 (12%)	31 (14%)	3 (6%)		12 (10%)	10 (10%)	2 (11%)	

^a^P-value from χ^2^ test of association

^b^Unpaid activities: housework/chores or being a students

In India, persons with disabilities were three times more likely to have access to state-sponsored pension support than persons without (3.1, 2.1–4.6), but access to non-state livelihoods support mechanisms including self-help groups, microfinance or cash for work schemes were similar for persons with and without disabilities (Table 6). In Cameroon, 96% of the sample did not have access to any state-sponsored benefits, and persons with disabilities were less likely than persons without to access non-state livelihoods support (e.g. self-help or microfinance groups run by non-state actors) (0.6, 0.4–0.9).

**Table 6:** Access to benefits and other livelihoods support.

	India^a^			Cameroon^a^		
	Cases n (%)	Controls n (%)	Age and Sex Adjusted OR (95% CI)	Cases n (%)	Controls n (%)	Age and Sex Adjusted OR (95% CI)
	n = 441	n = 288		n = 315	n = 184	
**State Sponsored Benefits**						
Pension	225 (51%)	67 (23%)	3.1 (2.1–4.6)	4 (1%)	2 (1%)	1.4 (0.4–5.8)
Other benefit	27 (6%)	3 (1%)	11.4 (3.4–38.0)	12 (4%)	4 (2%)	2.7 (1.0–7.2)
No benefits	189 (43%)	218 (76%)	Baseline	299 (95%)	178 (97%)	Baseline
**Non State Livelihoods support**						
Any support	106 (24%)	83 (29%)	0.9 (0.6–1.3)	146 (46%)	108 (59%)	0.6 (0.4–0.9)
Self Help Groups	76 (17%)	64 (22%)	0.8 (0.6–1.2)	130 (42%)	89 (49%)	0.7 (0.5–1.1)
Microfinance Groups	9 (2%)	6 (2%)	1.2 (0.4–3.8)	70 (22%)	53 (29%)	0.8 (0.5–1.2)
Cash for Work schemes	42 (10%)	31 (11%)	1.0 (0.5–1.8)	42 (13%)	30 (17%)	0.8 (0.5–1.5)
Other	1 (1%)	0	-	13 (4%)	5 (3%)	1.7 (0.7–4.2)

^a^Binary outcome variables with positive response presented–OR for each variable individually, adjusted for age and sex

‘-’Omitted due to small cell size

## Discussion

In this two-setting study, persons with disabilities in both settings were five times less likely to be working compared to age, sex and community matched controls without disabilities, and this relationship held across age groups, sex, marital status, and education level. Among persons with disabilities, key predictors of working were lower current age, being married and not having either a physical impairment in both settings, or multiple impairments in Cameroon. Even in the oldest age group of 65 and above, persons with disabilities were substantially less likely to be working than persons without disabilities.

The evidence of substantially lower participation in work among persons with disabilities compared to their non-disabled peers in this study corroborates the limited literature on the negative relationship between disability and access to livelihoods in LMICs [13, 44, 45]. The very limited available evidence from Cameroon and India specifically also corroborates our findings[46]. This reinforces the theorized pathway to poverty via barriers to decent work for persons with disabilities and their families and is contrary to Article 27 of the UNCRPD on the right of persons with disabilities to work on an equal basis as others[4].

Overcoming the gap in access to livelihoods between persons with and without disabilities is essential in view of the international mandates put forward by the SDA and UNCRPD. Labour market analyses in high-income countries have highlighted numerous components underpinning the employment gap between persons with and without disabilities. These include employer misconceptions about the productive capacity of persons with disabilities, insufficient environmental or physical accommodations to the individual’s needs, and increased reservation wages (the lowest wage at which a person will work) affected by unbalanced benefit policies that may dis-incentivise persons with disabilities to join or remain in the labour market[16, 17]. Such dimensions are less well explored or understood in LMICs and in the context of more complex livelihood mechanisms. One qualitative study by Palmer et al. (2015) in Vietnam cited low educational attainment and discrimination as the biggest barriers to both formal and informal work for persons with disabilities in that setting [47].

Possible mechanisms for promoting greater participation of persons with disabilities in livelihoods include improved access to social protection systems, healthcare, rehabilitation and assistive devices, education and vocational training [48, 49]. Furthermore, Article 2 of the UNCRPD outlines key obligations of governments to ensure equal opportunities for persons with disabilities. These commitments include establishing anti-discrimination laws, ensuring the accessibility of workplaces and, together with employers, providing reasonable accommodations to workers with disabilities[50]. However, evidence on both the availability and impact of these different interventions on improving access to livelihoods for persons with disabilities in LMICs is extremely minimal, and in urgent need of prioritisation [51].

Persons with disabilities were slightly more likely to be in the poorest socio-economic quartile in India, but in Cameroon, no differences in socio-economic status between groups was observed. This contrasts to prevailing literature that has shown an association between livelihoods and poverty, but is similar to findings shown in Afghanistan, Zambia and Rwanda[52, 53]. Reasons are unclear, but may reflect very high levels of poverty across the population making it more difficult to detect differences between groups. Similarly, it exposes the need to explore more nuanced measures of multidimensional poverty incorporating additional dimensions such as living standards and empowerment, in addition to measures of economic poverty.

The similarity in education and literacy levels between cases and controls in both settings, while seemingly contrasting to the growing evidence on the widespread exclusion of children with disabilities from school [54] should be interpreted in light of age of onset of disability. The majority of cases (51% in India and 77% in Cameroon) reported disability onset beyond school age. This serves as a reminder of the need to be cognisant of the potentially varying implications of disability as acquired at different time-points in the life-cycle.

Exploring predictors of access to livelihoods amongst persons with disabilities highlighted additional trends and the heterogeneity of the lived experience of disability. The finding that in India, women with disabilities were twice as likely not to be working as men with disabilities, supports the theorized ‘double discrimination’ experienced by women with disabilities [55, 56]. In contrast, in Cameroon no difference was observed by sex. This may be related to the high proportion of both cases and controls working in agrarian livelihoods in Cameroon (77%, compared with 50% in India), which may be less vulnerable to external barriers (e.g. accessibility, stigma) than those seeking livelihood opportunities through an employer or customer-facing business.

Our study found that marital status was strongly associated with disability and access to livelihoods. First, persons with disabilities were less likely to be married than persons without disabilities in both settings, which supports previous literature on disability stigma and societal misconceptions of asexuality of persons with disabilities [3, 57]. Second, among persons with disabilities, those who were married were more likely to work even after adjustment for confounders. This may be related to the psychological benefits of cohabiting with a partner, as opposed to being single, widowed or divorced, which has long been established to build human and social capital, improve psychological wellbeing and provide resilience [58]. Conversely, the reverse causality may hold, in that there may be increased likelihood of marriage amongst persons with disabilities who work. Further, ideally longitudinal, research is needed in this area.

Age of onset and type of functional limitation affected likelihood of access to livelihoods in different ways in the two settings. In terms of onset, amongst persons with disabilities, those who had acquired their disability aged fifty and above in India, and below five years of age in Cameroon were the least likely to be working. Physical limitations were associated with lower likelihood of working in both settings, alongside depression in India, and the presence of multiple limitations in Cameroon. As over half of persons with disabilities that worked in India and three quarters in Cameroon were small-scale farmers, the inherently physically-demanding nature of the predominant livelihood may explain why those with physical limitations in each setting were less likely to work. The finding that persons with depression were least likely to be working in India is in line with findings from a systematic review highlighting a strong association between common mental disorders and poverty–including but not restricted to economic poverty and employment–in LMICs [59].

Taken in aggregate, these findings substantiate arguments related to the heterogeneity of disability and the lived experiences of persons with disabilities, and highlights the importance of disability data disaggregation in research findings[16, 60, 61]. Moreover, in relation to the Sustainable Development Agenda, it necessitates responsiveness and reactivity even within the context of inclusive programme design to meet diverse needs, capacities and environmental contexts, and break down the barriers to engaging in sustainable livelihoods experienced by persons with disabilities in different contexts and settings.

The relatively high proportion of persons with disabilities receiving state-sponsored livelihood support in India, and non-state support in Cameroon, is encouraging, particularly in light of the evolving discourse and evidence related to social protection as a mechanism for mitigating and preventing poverty. Social protection will be most transformative when it addresses drivers of poverty and barriers to decent work, such as poor access to timely, affordable healthcare and quality education; however, the nature and impact of state and non-state supports in these contexts was not a focus of this research. The role of social protection in reducing poverty and improving livelihoods is a complex and nuanced research area, which deserves further attention in future studies[62].

### Strengths and limitations

Our primary dependent variable ‘working’ is a relatively narrow conceptualisation of livelihoods, and may miss some of the multiple productive activities that households and individuals may engage in, particularly in rural and informal economies [63–65]. Moreover, we are limited in our analyses by the cross-sectional nature of the data, despite our attempts to adjust for age of onset in relation to the outcome variables. In addition, the unexpectedly large burden of disability in the older age groups, while an important finding in itself, prevented us from achieving perfect age-sex matching of cases to controls.

In terms of strengths, this was a large population based case-control study in two settings assessing the quantitative relationship between disability and access to livelihoods. We used a comprehensive approach to measuring disability and assessed access to livelihood both between persons with and without disabilities, and amongst persons with disabilities.

## Conclusion

This study provides empirical evidence of the exclusion of persons with disabilities from livelihood opportunities in one district each of Cameroon and India. Moreover, the findings highlight the heterogeneity of that exclusion amongst persons with disabilities. This necessitates both adequately disaggregated quantitative data that fully reflects the spectrum of experiences by persons with disabilities in accessing livelihood opportunities, and appropriately reactive inclusive programmes that can meet diverse needs. The coverage of livelihood protective programmes and benefits in both settings was encouraging, and should be promoted within the context of sustainability and access to work.

## Supporting information

S1 File Final Questionnaire case control study, Telegu. (PDF)

S2 File Final Questionnaire case control study, English. (PDF)
