# Supplementary material for: Livelihood opportunities amongst adults with and without disabilities in Cameroon and India: A case control study
Source: PLoS One. 2018 Apr 9;13(4):e0194105. doi: 10.1371/journal.pone.0194105 (PMC5890974; doi:10.1371/journal.pone.0194105)
Supplement: S1 File — (PDF) [file pone.0194105.s001.pdf]

Andhra Pradesh Disability Study 2014-Case Control Questionnaire

ఆంధ్ర ప్రదేశ్ యొక్క అంగవైకల్య అధ్యయనం 2014-విషయ(కేస్) నియంత్రణ ప్రశ్నావళి

Cover Sheet
ముఖపత్రం

1 క్లస్టర్ సంఖ్య: Cluster No ☐☐

2 ఇంటి నెంబరు: House No ☐☐

3 వ్యక్తి ఐ .డి నెంబరు: Subject ID No. ☐☐☐

4 Subject Name
వ్యక్తి పేరు : ____________________________

5 Interviewer No. ☐☐☐
ఇంటర్వ్యూ చేసేవారి సంఖ్య:

6 Date (Day/Month/Year):
తేది (రోజు/ నెల /సంవత్సరం) : ____ / __ / ______

7 Language of Interview:
ఇంటర్వ్యూ భాష : x
1 = Telugu
తెలుగు
2 = Urdu
ఉర్దూ
3= English
ఇంగ్లీష్

8 Is study subject a case or control?
అధ్యయన విషయం కేస్ లేదా నియంత్రణ?
1 = Case (Person with a disability)
కేస్ (అంగవైకల్యం గల వ్యక్తి)
2 = Control
నియంత్రణ

9 What is the study subject's age (years)?
అధ్యయనం లో పాల్గొన్న వ్యక్తి వయస్సు (సంవత్సరాలలో)
☐

10 What is the study subject's gender (observe)
అధ్యయనం లో పాల్గొన్న వ్యక్తిలింగము (పరిశీలించండి)
1 = Male
పురుషుడు
2 = Female
స్త్రీ

11 Is the study subject the head of the household?
అధ్యయనం లో పాల్గొన్న వ్యక్తి ఇంటి యజమానా?
0 = No
కాదు
1 = Yes
అవును

12 Is there another person in this household who has already been interviewed?
ఈ ఇంటిలో ఇంతకుముందే ఇంటర్వ్యూ చేయబడిన మరియొక కేసు (వ్యక్తి) వున్నారా?
0 = No — if No GO To Q14
కాదు — ఒకవేళ మీసమాధానం కాదు అయితే 14వ పశ్న కి వెళ్ళండి
1 = Yes
అవును

13 If yes, what is that person's ID no (check participant card) ☐☐
అవును అయితే ఆ వ్యక్తి ఐ.డి సంఖ్య ఏమిటి? (పాల్గొన్నవారి కార్డు తనిఖి చేయండి)

14 Person interviewed:
ఇంటర్వ్యూ చేయబడిన వ్యక్తి:
1 = Direct interview with case/control
కేస్ (సమస్య కలిగిన వారు)/ నియంత్రణ (సాధారణ వ్యక్తులు) వ్యక్తితో నేరుగా ఇంటర్వ్యూ జరిగింది.
2 = Interview with proxy only
వారి తరఫున ప్రతినిధులు ఇంటర్వ్యూలో పాల్గొన్నారు.
ID of proxy respondent: సన్నిహితుని ఐ.డి: ☐☐
ID of proxy respondent: సన్నిహితుని ఐ.డి: ☐☐
3 = Interview with proxy and case/control together
కేస్ (సమస్య కలిగిన వారు)/ నియంత్రణ (సాధారణ వ్యక్తులు) ప్రతినిధులను కలిపి ఇంటర్వ్యూ చేయడం జరిగింది .

A SOCIO-ECONOMIC QUESTIONS
సామాజిక- ఆర్థిక ప్రశ్నలు (కుటుంబ సంబంధపాత కేసు)

I would first like to check your height and your weight.
నేను ముందుగా మీ ఎత్తు మరియు బరువు పరిశీలించాలని అనుకుంటున్నాను

1. Interviewer fill in:
ఇంటర్వ్యూయర్ ఇందులో నింపాలి

1=Weight Measured
బరువు
2 =Weight not measured (wheelchair)
బరువు తీసుకోలేదు (విల్ చైర్)
3=Weight not measured (other reason) Specify
బరువు తీసుకోలేదు (ఇతర కారణాలు) తెలియజేయండి________________________________

2 Weight in Kilograms
బరువు కిలోగ్రాములలో
[ ][ ] [ ][ ]

3. Interviewer fill in:
ఇంటర్వ్యూయర్ ఇందులో నింపాలి

1=Height measured standing
నిలబడినప్పుడు ఎత్తు కొలత
2=Height measured lying down
పడుకున్నపుడు ఎత్తు కొలత
3 =Height not measured Reason
ఎత్తు కొలత తీసుకోలేదు కారణం__________________________________

4 Height in Centimeters
ఎత్తు సెంటి మీటర్ లలో
[ ][ ] [ ][ ]

I am now going to ask you a few questions about your household
నేను మీ కుటుంబానికి సంబంధించి కొన్ని ప్రశ్నలను అడగబోతున్నాను.

Note to Interviewer: If there is more than one case or control in the household, this section should be asked to the first person interviewed ONLY
ఇంటర్వ్యూ చేసేవారికి సూచన : ఒకవేళ గృహంలో ఒకటికంటే ఎక్కువ కేసులున్నచో ఈ విభాగంలో మొదటికేసును గురించి మాత్రమే అడగాలి.

1 How many rooms are there in your household (excluding bathrooms, kitchens, balconies and corridors)?
మీ ఇంటిలో ఎన్ని గదులు ఉన్నాయి? (స్నానాలగదులు, వంటగదులు, బాల్కనీలు, కారిడార్లు మినహాయించి)

2 What is the main source of lighting in your household?
మీ ఇంట్లో దీపాలకు మూలాధారం ఏమిటి?
ప్రధాన విద్యుత్ జనరేటర్ / బ్యాటరీ /ఇన్ వర్టర్

1 = Mains power
ప్రధాన విద్యుత్
2 = Generator/battery/inverter
జనరేటర్ / బ్యాటరీ /ఇన్ వర్టర్
3 = Kerosene/oil/petrol lamps
కిరోసిన్/ నూనె/ పెట్రోమాక్స్ లైట్
4 = Candles
కొవ్వొత్తులు
5 = No lighting
దీపాలు లేవు
6 = Other, specify:
ఇతరములు తెలియజేయండి:

3 Does any member of your household own the following (in working order):
మీకుటుంబంలో ఎవరైనా వీటిని కలిగివున్నారా? (పనిచేస్తున్న స్థితిలో)

	0 = No<br>కాదు	1 = Yes<br>అవును		0 = No<br>కాదు	1 = Yes<br>అవును
a Radio/HiFi/Stereo<br>రేడియోస్టీరియో/హై ఫై/	0	1	j Washing machine<br>వాషింగ్ మిషన్	0	1
b TV/VCR/DVD	0	1	k Sewing machine	0	1

టిడి.వి.డి/ఆర్.సి.వి/వి.			కుట్టు మిషన్			
c Fridge/Freezer<br>ఫ్రిజ్‌ఫ్రీజర్/	0	1	l Air conditioner<br>ఎయిర్ కండిషనర్	0	1	
d Mobile phone<br>మొబైల్ ఫోన్	0	1	m Bicycle<br>సైకిల్	0	1	
e Cupboard<br>కప్ బోర్డ్	0	1	n Stove with gas<br>గ్యాస్ స్టవ్	0	1	
f Sofa set/armchair<br>సోఫాసెట్‌కకుర్చీపడ /	0	1	o Stove with electric<br>ఎలక్ట్రిక్ స్టవ్	0	1	
g Table<br>టేబుల్	0	1	p Computer<br>కంప్యూటర్	0	1	
h Motor vehicle incl cars<br>మోటార్ వాహనము కారులతో కలిపి	0	1	q Kerosene Gas Stove<br>కిరోసిన్ స్టవ్	0	1	
i Motorbike<br>మోటార్ సైకిల్	0	1	r wooden stove<br>కట్టెల పొయ్యి	0	1	

4 What is the ownership of your household's dwelling?

మీ కుటుంబం యొక్క నివాసస్థలం యాజమాన్యం ఏమిటి?

1= House owned by household
కుటుంబం యొక్క స్వంత ఇల్లు

2= Rented house
అద్దె ఇల్లు

3= Government owned house
ప్రభుత్వ ఇల్లు

4= Other, specify:
ఇతరములు, తెలియజేయండి:____________________

B. Water and Sanitation
నీరు మరియు పారిశుద్ధ్యము

I would now like to ask you a few questions about water and sanitation:
నేను మిమ్మల్ని నీరు మరియు పారిశుద్ధ్యము గురించి కొన్ని ప్రశ్నలు అడగాలని అనుకుంటున్నాను

Note to Interviewer: Questions **1**,**2**, **7**, **8** and **9** should only be answered once for each household. If there is more than one case or control in the household, this section should be asked to the first person interviewed ONLY

ఇంటర్వ్యూ చేసే వారికి గమనిక - 1,2,7,8మరియు9ప్రశ్నలకు కుటుంబములో ఒక్కరే సమాధానం ఇవ్వాలి చోఒకవేళ గృహంలో ఒకటికంటే ఎక్కువ కేసులున్న . మొదట ఇంటర్వ్యూ చేసిన కేసు గురించి మాత్రమే నింపాలి.

1 What kind of toilet facilities do members of your household usually use
మీ కుటుంబసభ్యులు సాధారణంగా ఎటువంటి మరుగుదొడ్లను ఉపయోగిస్తారు?

1 = Flush toilet
ఫ్లష్ టాయిలెట్

2 = Traditional latrine
సాంప్రదాయ మరుగుదొడ్డి

3 =Ventilation improved
గాలి ప్రసరణ మెరుగుపడినది
మరుగు గుంత

4 = Bowl/Bucket
పాత్రబకెట్ /

5 = Other, Specify:
ఇతరములు)తెలియజేయండి.(______

6 = No toilet
మరుగు దొడ్డి లేదు

2 Do you share this facility with other households?
దీనిని ఇతర గృహస్థులు అందరూ వాడతారా ?

1 = Used only by your household
మీకుటుంబసభ్యులచేత మాత్రమే వాడబడుతుంది

2= Shared with other households
ఇతర గృహస్థులచేత కూడా వాడబడుతుంది.

3 = Public/ Communal/ Community Latrine

బహిరంగ/వర్గ /సాముహిక మరుగుదొడ్డి

3 Do you use the same toilet facility as other members of your household?
మీ కుటుంబసభ్యులు వాడిన మరుగుదొడ్డినే మీరు వాడతారా?

0 = No లేదు

1 = Yes అవును **If YES, go to Q6** అవును అయితే 6వ ప్రశ్నకు వెళ్ళుము

4 Why do you use a different toilet facility from other members of your household (main reason)
మీకుటుంబం లోని ఇతరసభ్యులవలే కాకుండా మీరు ఎందుకు వేరే సౌకర్యంగల మరుగుదొడ్డిని ఉపయోగిస్తున్నారు (ముఖ్య కారణం(

1 = It would be physically impossible
అది శారీరకంగా అసాధ్యం

2 = I'm not allowed/others would not like it
నాకు అనుమతి లేదు ఇతరులు ఇష్టపడరు/

3 = I might face verbal or physical abuse
నేను దూషణ లేదా శారీరిక వేధింపులను ఎదుర్కోవలసి రావచ్చు

4 = I would be embarrassed
నాకు మొహమాటం

5 = Other (specify) ____________________
ఇతరములు (తెలియజేయండి)

5 What kind of toilet facility do you usually use?
మీరు ఎటువంటి రకం మరుగుదొడ్డిని ఉపయోగిస్తారు?

1 = Flush toilet
ఫ్లష్ టాయిలెట్

2 = Traditional latrine
సాంప్రదాయ మరుగుదొడ్డి

3 =Ventilation improved
గాలి ప్రసరణ మెరుగుపడినది
మరుగు గుంత

4 = Bowl/Bucket
పాత్రబకెట్ /

5 = Other, Specify:
ఇతరములు)తెలియజేయండి.(______

6 = No toilet
మరుగు దొడ్డి లేదు

6 Are you usually able to use the toilet facility without you or your clothes coming into contact with feaces
మీరుసాధారణంగా మీకుగాని, మీబట్టలకుగాని మలమూత్రాదులు అంటకుండా మరుగుదొడ్డిని ఉపయోగించ గలుగుతున్నారా?

0 = No కాదు

1 = Yes అవును

7 What is the main source of drinking water for members of your household?
మీయొక్క కుటుంబసభ్యులకు త్రాగునీటికి ముఖ్య నీటివనరు ఏది?

1 = Private pipeline
ప్రైవేటు కుళాయి

2 = Private well
ప్రైవేటు బావులు

3 = Public taps/standpipe
బహిరంగకుళాయిలుచేతిపంపులు/

4 = Public well
బహిరంగ బావులు

5 = Neighbors
ఇరుగుపొరుగు నుండి

6 = Water vendor
నీటి విక్రయదారులు

7 = Spring
నీటిబుగ్గలు

8 = River/stream/lake
నదిసరస్సులు/కాలువలు/

9 = Rainwater
వర్షపు నీళ్ళు

10 = Other, specify:
ఇతరములు, తెలియజేయండి:______________________

8 How long does it take to go there, get water and come back? minutes
అక్కడకు వెళ్ళి నీళ్ళు తీసుకొనిరావడానికి ఎంత సమయం పడుతుంది? నిమిషాలు

9 Where do members of your household normally bath?
మీ కుటుంబసభ్యులు ఎక్కడ స్నానంచేస్తారు?

1 = Surface water (eg. Pond, river, sea)
ఉపరితల నీరు ఉ)దాచెరువు :, నది ,సముద్రము(

2= Pump or standpipe stored outside compound
ప్రహారీ బయట నిల్వచేయబడిన పంపు లేదా చేతి పంపు

3= Piped or stored water inside the house or
ఇల్లు లేక ప్రహారీ లోపల పంపు లేదా నిల్వ నీళ్ళు

10 Do you collect water for drinking?

మీరు త్రాగు నీటిని **సేకరిస్తారా?** 0 = No **If NO, go to Q15**

లేదు లేదు, అయినచో 15 వ ప్రశ్నకు వెళ్ళండి

1 = Yes **If YES, go to Q17**

అవును అవును అయితే, 17 వ ప్రశ్న కు వెళ్ళండి

11 Do you collect drinking water from the same source as other members of your household?

మీరు త్రాగు నీటిని మీ ఇతర కుటుంబ సభ్యులు సేకరించే ప్రాంతం నుండి తెచ్చారా?

0 = No లేదు 1 = Yes అవును

12 How long does it take to go there, get water and come back? minutes

అక్కడికి వెళ్లి నీళ్లు తీసుకొని వచ్చుటకు ఎంతసమయం పడుతుంది? నిమిషాలు.

13 From what source do you usually collect drinking water?

సాధారణముగా మీరు ఎక్కడి నుండి త్రాగునీటినితెచ్చుకొంటారు?

1 = Private pipeline
ప్రైవేటు కుళాయి

2= Private hand pump/tap
ప్రైవేటు చేతి పంపు/ కుళాయి

3 = Private well
ప్రైవేటు బావులు

4 = Public hand pump/tap
బహిరంగ చేతి పంపు/ కుళాయి

5 = Public well
బహిరంగ బావులు

6 = Neighbours
ఇరుగుపొరుగు నుండి

7 = Water vendor
నీటి విక్రయదారులు

8 = Spring
నీటిబుగ్గలు

9 = River/stream/lake
నది/కాలువలు/సరస్సులు

10 = Rainwater
వర్షపు నీళ్లు

11 = Other, specify:
ఇతరములు, తెలియచేయండి_____________________________________

14 Is this the same source as the water you use for bathing?

మీరు స్నానానికి ఉపయోగించే నీటికి ఇదే ఆధారమా?

0 = No లేదు 1 = Yes అవును

15 Could you collect water from the same source used by

మీరు నీటిని మీ ఇతర కుటుంబ సభ్యులు ఉపయోగించిన అదే

నీటివనరు నుండే వాడుకోగలరా?

0 = No కాదు

1 = Yes **If YES, go to Q17**

అవును అవును, అయినచో 16వ ప్రశ్నకు వెళ్ళండి

16 If no, why not?

సమాధానము 'లేదు' అయిన, ఎందుచేత?

1 = It would be physically impossible
ఇది భౌతికంగా అసాధ్యము

2 = I'm not allowed/others would not like it
నాకు అనుమతి లేదు/ ఇతరులు ఇష్టపడరు

3 = I might face verbal or physical abuse
నేను దూషణ లేదా శారీరక వేధింపులను ఎదుర్కొనవలసి రావచ్చును

4 = I would be embarrassed
నాకు మొహమాటము

5 = Other (specify)
ఇతరములు (తెలియచేయండి)

17 Are you able to access drinking water at home without assistance?

ఇంట్లో మీరు ఇతరుల సహాయం లేకుండా తాగునీరునిపొందగలుగుతున్నారా?

0 = No లేదు

1 = Yes అవును

18 Have you had diarrhea (4 or more loose stools within 24 hours) anytime in the past four weeks?

గత నాలుగు వారాలలో మీరు ఎప్పుడైనా అతిసారవ్యాధికి లోనయ్యారా? (24 గంటలలో 4 సార్లు వదులుగా మలం విసర్జించడం)

0 = No లేదు

1 = Yes అవును

C. Marital status, literacy and education (PARTICIPANTS 18 YEARS AND ABOVE ONLY)

వైవాహిక స్థితి, అక్షరాస్యత మరియు విద్య (18సం.లు ఆపై వయసువారు మాత్రమే పాల్గొనాలి)

Now I would like to ask you a few questions about your living status and education

ఇప్పుడు నేను మిమ్మల్ని మీ జీవన స్థాయి మరియు విద్యల గురించి కొన్ని ప్రశ్నలు అడగాలని అనుకుంటున్నాము.

1 What is your marital status?

మీ యొక్క వైవాహిక స్థితి ఏమిటి?

1 = Married or living together
వివాహితులు లేదా సహజీవనం
2 = Divorced/separated
విడాకులుపొందారు/విడిపోయారు
3 = Widowed
వితంతవు
4 = Never married/living together
అవివాహిత/సహజీవనం

2 Can you read well, a little or not at all?
మీరు బాగా చదవగలరా, కొంచెమా, లేక అస్సలు చదవలేరా?

1 = Well బాగా
2 = A little కొంచము
3 = Not at all అస్సలు లేదు

3 Have you ever attended School
మీరు ఎప్పుడైనా పాఠశాలకు వెళ్ళారా?

0 = No లేదు — **If NO, go to Q6** లేదు, అయినచో 6వ ప్రశ్నకు వెళ్ళండి
1= Yes అవును

4 What is the highest level of education
మీ యొక్క అత్యధిక విద్యార్హత ఏమిటి?

1 = Primary ప్రాథమిక
2 = Secondary మాధ్యమిక
3 = High School ఉన్నత పాఠశాల
4 = University విశ్వవిద్యాలయం
5= Religious School మతపరమైన పాఠశాల
6= No education చదువుకోలేదు

5 What was the highest grade that you completed? ☐ **Go to Q7** If University code 15
మీరు పూర్తి చేసిన అత్యధిక తరగతి ఏమిటి? 7 వ ప్రశ్నకు వెళ్ళండి యూనివర్సిటీ అయితే 15 కోడ్ వేయండి

6 What is the main reason why you did not receive formal education?
మీరు ప్రాథమిక విద్య పొందలేక పోవుటకు గల కారణం ఏమిటి?

1 = absence of school
పాఠశాల లేకపోవుట
2 = Lack of money
డబ్బు లేకపోవుట
3 = Needed to work
పని చేయవలసి వచ్చుట
4 = Education not very useful
విద్య అంత ఉపయోగం కాదు
5 = Being disabled was refused
వైకల్యము చేత తిరస్కరించబడ్డార
6 = Don't like school
పాఠశాల ఇష్టం లేదు
7 = Too much household work
చాలా ఇంటిపని
8 = Family does not allow
కుటుంబం అనుమతించదు
9 = No transport
రవాణా సౌకర్యం లేదు
10 = Other - specify
ఇతరము, తెలియచేయండి

Ask this question about head of the household (if he/she is NOT the study subject)
కుటుంబ యజమానిని ఈ ప్రశ్న అడగండి (అధ్యయనం లోని వ్యక్తి కాని)

7 What is the highest level of education the head of your household completed?
(పేరు) పూర్తిచేసిన అత్యధిక విద్యార్హత ఏమిటి?

1 = Primary ప్రాథమిక
2 = Secondary మాధ్యమిక
3 = High School ఉన్నత పాఠశాల
4 = University విశ్వవిద్యాలయం
5= Religious School మతపరమైన పాఠశాల
6= No education చదువుకోలేదు

8 What was the highest grade that the head of your household completed?
మీ ఇంటి పెద్ద పూర్తిచేసిన అత్యధిక తరగతి ఏమిటి ?

9 . Can the head of your household read well, a little or not at all?
ఇంటి పెద్ద బాగా చదవగలరా, కొంచమా, అస్సలు లేదా? 1 = Well బాగా 2 = A little కొంచము 3 = Not at allఅస్సలు లేదు

D. Education questions (CASES/CONTROLS AGED 17 YEARS AND BELOW ONLY)
విద్యాసంబంధిత ప్రశ్నలు (17 సం.లు మరియు ఆ లోపు వయసు కేసు వారికి మాత్రమే)

Note to interviewer: If answered by proxy replace "are you" with "is [name]"
ఇంటర్వ్యూ చేసే వారికి **గమనిక**: సన్నిహితుడు సమాధానమిస్తుంటే 'మీరు' ని అతని (పేరు)తో మార్చండి.

1 Are you currently enrolled in school? 0 = No **If NO, go to Q7**
మీరు ప్రస్తుతం పాఠశాలలో చేరారా? 0 = లేదు లేదు అయినచో 7 వ ప్రశ్నకు వెళ్ళండి
1 = Yes అవును

2 Are you enrolled in the same grade as other children your age?
మీ తోటివయసు పిల్లలతో అదే తరగతిలో చేరారా?
1= Yes
అవును
2= No, lower grade than other children my age
లేదు, నా సమవయస్కులకన్నా క్రింది తరగతి
3= No, a higher grade than other children my age
లేదు, నా సమవయస్కులకన్నా పై తరగతి

3 Is the school you are in a mainstream/regular school or special school?
మీరు వున్న పాఠశాల ప్రధానమైన/సాధారణ పాఠశాల లేదా ప్రత్యేక పాఠశాలా?
1 = Mainstream/regular
ప్రధానమైన/సాధారణ పాఠశాల
2 = Special school
ప్రత్యేక పాఠశాల
3 = Integrated
సమగ్రమైన
4 Mainstream with special/extra
ప్రత్యేక/అదనపు తరగతులు కలిగిన ప్రధాన పాఠశాల

4 In the last month of school, how many days did you miss? Days
గత నెలలో ఎన్నిరోజులు మీరు ఎన్ని రోజులు పాఠశాలకు వెళ్ళలేకపోయారు? రోజులు

5 Have you ever repeated a grade at school 0 = No **If NO, go to Q11**
మీరు ఎప్పుడైనా అదే తరగతిని మళ్ళీ చదివారా? 0 = లేదు లేదు అయినచో 11 వ ప్రశ్నకు వెళ్ళండి
1 = Yes అవును

6 If yes, how many times have you repeated a grade at school ☐ **Go to Q11**
మీ జవాబు అవును అయినచో, పాఠశాలలో ఒకే తరగతిని ఎన్ని సార్లు చదివారు? ప్రశ్న 11 కి వెళ్ళండి

7 If not currently enrolled, have you ever 0 = No **If NO, go to Q10**
ప్రస్తుతం చేరని పక్షంలో, ఎప్పుడైనా పాఠశాలకు వెళ్లావా? 0 = లేదు లేదుఅయినచో 10వ ప్రశ్నక వెళ్ళండి
1 = Yes అవును

8 What is the highest level of education
మీరు పూర్తి చేసిన అత్యధిక **విద్యాస్థాయి** ఏది
1 = Primary ప్రాధమిక
2 = Secondary మాధ్యమిక
3 = High School ఉన్నత పాఠశాల
4 = University విశ్వవిద్యాలయం
5= Religious School మతపరమైన పాఠశాల

9 What was the highest grade that you completed?
మీరు పూర్తిచేసిన అత్యధిక తరగతి ఏది?

10 If you have never attended/are currently not attending school, what is the main reason?
ఒకవేళ మీరు ఎప్పుడు పాఠశాలకు హాజరు కాకపోవుటకు/ప్రస్తుతం వెళ్లకుండా ఉండుటకు గల ముఖ్యకారణములు ఏమిటి?

1 = Not enough money
తగినంత డబ్బు లేక
2 = Lack of interest to go to school
పాఠశాల వెళ్లుటకు ఆసక్తి లేక
3 = Lack of school nearby
దగ్గరలో పాఠశాల లేక
4 = Nearby school not accessible
దగ్గరలోని పాఠశాల అందుబాటులో లేక
5 = Illness (<1 month)
అనారోగ్యం (ఒక నెల వరకు)
6 = Illness (> 1 month)
అనారోగ్యం (ఒక నెల పైన)
7 =Attendance refused by school
హాజరు లేకపోవటం వలన పాఠశాల తిరస్కరించుటచేత
8 = Negative attitudes of other students
ఇతర విద్యార్థుల ప్రతికూల వైఖరి
9 = Negative attitudes of teachers
ఉపాధ్యాయుల ప్రతికూల వైఖరి
10 = Lack of accessible resources to assist child
పిల్లల సహాయానికి వనరుల అందుబాటులోకొరత
11 = Child works
బాల కార్మికుడు
12= Other, Specify:
ఇతరములు, తెలియచేయండి:___________________

Go to Section F
సెక్షన్ f కి వెళ్ళండి

CASES AND CONTROLS (5-17) CURRENTLY ENROLLED IN SCHOOL ONLY
ప్రస్తుతం పాఠశాలలో (కేసులు మరియు నియంత్రణలు) చేరిన పిల్లల(5-17) కోసము మాత్రమే

Q11 If you currently attend school, how often do the following situations happen to you?
ఇప్పుడు మీరు పాఠశాలకు వెళ్తూ ఉంటే, ఎంత తరచుగా ఈ పరిస్థితులను మీకు జరుగుతున్నాయి?

	Always ఎల్లప్పుడు	Sometimes అప్పుడప్పుడు	Never ఎప్పుడు కాదు	Don't know తెలియదు
A. If you have a problem at school there are teachers willing to help you<br>ఒకవేళ మీకు పాఠశాలలో సమస్య కలిగినప్పుడు అధ్యాపకులు సహాయానికి సుముఖంగా ఉంటారు	1	2	3	4
B. If you have a problem at school there are friends to help you<br>ఒకవేళ మీకు పాఠశాలలో సమస్య కలిగినప్పుడు తోటి విద్యార్థులు సహాయం చేస్తారు	1	2	3	4
C. If your friends have a problem at school they come to you for help<br>ఒకవేళ పాఠశాలలో మీ స్నేహితులకు సమస్యకలిగినపుడు మీ వద్ద సహాయం కోసం వస్తారు	1	2	3	4
D. You have friends that you play with at breaktimes<br>విరామ సమయాలలో ఆడుకోవటానికి మీకు స్నేహితులు ఉన్నారు	1	2	3	4
E. Your friends look up to you as a leader<br>మిమ్మల్ని నాయకునిగా మీ స్నేహితులు భావిస్తారు	1	2	3	4
F. Children hit, hurt or say nasty things to you<br>విద్యార్థులు కొట్టడం గాయపరచడం లేదా అసభ్యమైన మాటలను మాట్లాడటం వంటివి మీతో చేస్తారు.	1	2	3	4
G. You are included in lessons and school activities<br>అధ్యాపకులు కొట్టడం గాయపరచడం లేదా అసభ్యమైన మాటలను మాట్లాడటం వంటివి మీతో చేస్తారు.	1	2	3	4
H. Your school has the right materials to help you learn<br>పాఠ్యాంశాలు మరియు పాఠశాల కార్యకలాపాలలో మిమ్మల్ని కలుపుకుంటారు.	1	2	3	4
I. Your school has the right materials to help you learn<br>మీ పాఠశాల మీరు నేర్చుకొనుటకు సహాయపడే వస్తుసామగ్రి కలిగియుంది.	1	2	3	4

ఒకవేళ కంట్రోల్స్ అయితే సెక్షన్ f కి వెళ్ళండి ఒకవేళ case అయితే కొనసాగించండి

Q **<u>CASES</u> (5-17) CURRENTLY ENROLLED IN SCHOOL ONLY**

ప్రస్తుతం పాఠశాలలో(5-17) చేరిన కేసుల కోసము మాత్రమే

12 I want to know more about your school and whether it is adapted for your needs
మీ అవసరాలకు తగిన విధంగా మీ పాఠశాల వుందో లేదో తెలుసుకోవాలని నేను అనుకుంటున్నాను

	Yes అవును	No కాదు	Not Applicable వర్తించదు
A. Do you get extra time to complete work or tests<br>పని లేదా పరీక్షలలో అధిక సమయం మీకు లభిస్తోందా ?	1	2	3
B. Does the teacher teach in a way that makes it easy for you to learn and understand<br>మీకు సులభంగా అర్ధమయే పద్ధతిలో అధ్యాపకులు బోధిస్తారా?	1	2	3
C. Do you get extra lessons?<br>మీరు అదనపు పాఠాలు పొందుతారా?	1	2	3
D. Are teaching aids adapted for you eg. Are pictures used to supplement text and what is spoken about<br>మీకు తగినట్లుగా బోధనోపకరణాలు ఉన్నాయా? ఉదా. చెప్పే వాటికి సరైన చిత్రపటాలు, వాడుతారా?	1	2	3
E. Does another person in the classroom help you?<br>తరగతిగదిలో మీ సహాయానికి ఇంకొక వ్యక్తి ఉన్నారా?	1	2	3
F. Is the class environment adapted for you eg. Lighting, or space is made for you to sit at the front?<br>తరగతి పరిసరాలు మీకు తగినవిధంగా ఉన్నాయా? ఉదా. వెలుగు లేదా ముందు బాగంలో కూర్చోవటానికి సరైన స్థలం	1	2	3
G. Are communication devices used to help you eg. Communication boards, software, voice output devices<br>మీకు సహాయపడటానికి ప్రసార మాధ్యమాలు ఉపయోగిస్తున్నారా? ఉదా. సమాచార పట్టికలు, సాఫ్ట్ వేర్, శబ్ద పరికరాలు	1	2	3
H. Is text put in Braille or large font, or audio taped?<br>పుస్తకాంశాలు బ్రెయిలీ, పెద్ద అక్షరాలు లేదా ధ్వనిముద్రికా మాధ్యమంగా కాని ఉన్నాయా?	1	2	3
I. Are hearing or visual aids used<br>దృశ్య శ్రవణోపకరణాలు వదబడుతున్నాయా	1	2	3
J. Does someone use sign language with you?<br>మీతో ఎవరైనా సైగల భాషలో మాట్లాడుతారా?	1	2	3
K. Is the playground accessible?<br>క్రీడాస్థలం అందుబాటులో ఉందా?	1	2	3
L Is there an accessible toilet?<br>అక్కడ అందుబాటులో మరుగుదొడ్డి ఉందా?	1	2	3

13 At school are you able to use the same toilet facility as

పాఠశాలలో ఇతర విద్యార్ధులవలె అదే మరుగుదొడ్డి వసతి వాడగల్గుతున్నారా?

0 = లేదు No

1 = Yes **If YES, go to Q15**

1 = అవును అవును, అయినచో Q14 వెళ్ళండి

14 If no, what is the main reason why not?

సమాధానము 'కాదు' అయిన, ఎందుకు కాదు, ముఖ్యకారణము ఏమిటి?

1 = It would be physically impossible
అది శారీరకంగా అసాధ్యము

2= I could not use it without getting myself or my clothes soiled
నేనుకాని నా బట్టలుకాని మురికి అవకుండా వాడటం సాధ్యం కాదు

3 = I'm not allowed/others would not like it
నాకు అనుమతి లేదు/ ఇతరులు ఇష్టపడరు

4 = I might face verbal or physical abuse
నేను దూషణ లేదా శారీరక వేధింపులను ఎదుర్కొనవలసి రావచ్చును.

5 = I would be embarrassed
నాకు మొహమాటము

6 = Other (specify)
ఇతరములు (తెలియజేయండి)____________________

15 At school are you able to access drinking water from the same source as other pupils?

పాఠశాలలో ఇతర విద్యార్ధులువాడే నీటివనరునుండి తాగునీరు పొందగలరా?

0 = No

0 = లేదు

1 = Yes **If YES, go to Q17**

1 = అవును అవును, అయినచో Q17 వెళ్ళండి

16 If no, what is the main reason why not?

సమాధానము 'కాదు' అయిన, ఎందుకు కాదు, ముఖ్యకారణము ఏమిటి?

1 = It would be physically impossible
అది శారీరకంగా అసాధ్యము

2 = I'm not allowed/others would not like it
నాకు అనుమతి లేదు/ ఇతరులు ఇష్టపడరు

3 = I might face verbal or physical abuse
నేను దూషణ లేదా శారీరక వేధింపులను ఎదుర్కొనవలసి రావచ్చును

4 = I would be embarrassed
నాకుమొహమాటము

5 = Other (specify)
ఇతరములు (తెలియజేయండి)________________________

17 At school are you able to wash your hands at the same place as other pupils?

పాఠశాలలో ఇతర విద్యార్ధులు చేతులు కడుగుకొనే ప్రదేశంలోనే మీరూ కడుగుకోగలుగుతున్నారా?

0 = No లేదు

1 = Yes **If YES, GO TO Section F**

1 = అవును అవును, అయినచో F విభాగానికి వెళ్ళండి

18 If no, what is the main reason why not?

సమాధానము 'కాదు' అయిన, ఎందుకు కాదు, ముఖ్యకారణము ఏమిటి?

1 = It would be physically impossible
అది శారీరకంగా అసాధ్యము

2 = I'm not allowed/others would not like it
నాకు అనుమతి లేదు/ ఇతరులు ఇష్టపడరు

3 = I might face verbal or physical abuse
నేను దూషణ లేదా శారీరక వేధింపులను ఎదుర్కొనవలసి రావచ్చును

4 = I would be embarrassed
నాకు మొహమాటము

5 = Other (specify)
ఇతరములు

(తెలియజేయండి)______________________

GO TO Section F సెక్షన్ F కి వెళ్ళండి

E Livelihood questions (CASES/CONTROLS AGED 18 YEARS AND ABOVE ONLY)
జీవనోపాధి సంబంధిత ప్రశ్నలు (18సం.లు ఆపై వయసు కేసులు/నియత్రణలకు మాత్రమే)

I would now like to ask you some questions about work
నేను ఇప్పుడు మీ పనికి సంబంధించి కొన్ని ప్రశ్నలు అడగాలని అనుకుంటున్నాను.

1 Other than domestic work in the household Have you done any work in the last seven days?
ఇంట్లో గృహసంబంధిత పనులు కాకుండా గత ఏడురోజులలో ఏదైన ఇతర పని చేశారా?

0 = No కాదు

1= Yes అవును **If YES, Go to Q4** అవును అయితే Q4 వెళ్ళండి

2 Although you did not work in the last seven days, do you have any job or business from which you were absent for leave, illness, vacation, or any other such reason?
గత ఏడు రోజులుగా ఏపనికి వెళ్లకపోయినా, మీరు సెలవు చేతకానీ, అనారోగ్యం చేతకానీ లేదా ఏ ఇతర కారణం చేత వెళ్ళలేకపోయిన వ్యాపారము లేదా పని మీకు ఉందా?

0 = No కాదు

1= Yes అవును **If YES, Go to Q4** అవును అయితే Q4 వెళ్ళండి

3 Have you done any work in the last 12 months?
గత 12 నెలలుగా మీరు ఏదైనా పని చేశారా?

0 = No **If NO, go to Q8**
0 = లేదు లేదు అయితే Q8 వెళ్ళండి
1= Yes అవును

4 What is your occupation, that is, what kind of work do you mainly do?
మీ వృత్తి ఏమిటి? అనగా ఎటువంటి పనిని మీరు ముఖ్యంగా చేస్తారు?

5 In this work do you : work on your own/household's business (e.g. Shopkeeper, taxi driver, carpenter, barber) or work for someone who is not a member of your household (e.g. enterprise, company, government/other individual) or work on farm owned/rented by yourself or household member
ఈ పనిలో మీరు : సొంతముగా పని చేస్తారు/కుటుంబవ్యాపారాన్ని (ఉదా: దుకాణదారు, టాక్సీ డ్రైవర్, వడ్రంగి, క్షురకుడు) లేదా మీ కుటుంబసభ్యుడు కాని ఇతరులకోసం పనిచేస్తారు (ఉదా: వ్యాపారము, సంస్థ, ప్రభుత్వ/ లేదా వ్యక్తిగత) లేదా(సొంత వ్యవసాయం / కౌలుదారుగా పనిచేయటం లేదా కుటుంబసభ్యులు)

1 = own/household business సొంత/కుటుంబవ్యాపారం

2 = non-household member కుటుంబసభ్యులు కాదు

3 = farm owned/rented by household సొంత వ్యవసాయం/ కుటుంబంచే కౌలు తీసుకొన్నది

6 Do you usually work throughout the year, or do you work seasonally, or only once in a while?
మీరు సాధారణముగా సంవత్సరపొడవునా పనిచేస్తారా లేదా కాలానుగుణంగా పని చేస్తారా లేదా ఎప్పుడైనా ఒకసారి పని చేస్తారా?

1= Throughout the year
సంవత్సర పొడవునా

2 = Seasonally/part of the year
కాలానుగుణంగా/ సంవత్సరంలో కొంతభాగం

3 = Once in a while
ఎప్పుడైనా ఒకసారి

7 Are you paid in cash or kind for this work or are you not paid at all?
ఈ పని కోసం మీకు నగదు చెల్లిస్తారా లేదా వస్తు రూపంలో చెల్లిస్తారా లేదా అస్సలు చెల్లించరా?

1 = Cash only
నగదు మాత్రము

2 = Cash and kind
నగదు/వస్తు రూపంలో

3 = In kind only
వస్తు రూపంలో మాత్రమే

4 = Not paid
అస్సలు చెల్లించరు

Q9 కి వెళ్ళండి

8 If not working, what is the main reason ?
చేయకపోవటానికి గల ముఖ్య కారణం ఏమిటి?

1= Student
విద్యార్థి

2=Childcare/duties/work inside the house
పిల్లల్ని చూసుకొనుట/బాధ్యతలు/ఇంటిలో పని

3= Too old / retired
వృద్ధాప్యం/ పదవీ విరమణ చేత

4= Incapable of working, physically
శారీరకంగా పని సామర్ధ్యం లేక

5= Incapable of working, mentally
మానసికంగా పని సామర్ధ్యం లేక

6= Nobody would give me a job because I am disabled
నా వైకల్యకారణంగా ఎవరు పని ఇవ్వరు

7= Long illness (>1 month)
సుదీర్ఘ అనారోగ్యం (>నెలకంటే ఎక్కువ)

8= I am looking for my first job
నేను నా మొదటి ఉద్యోగంకోసం చూస్తున్నాను

9= No jobs opportunities in the area
ప్రాంతంలో ఉద్యోగావకాశాలు లేవు

10= Quit/suspended from job
వదిలిపెట్టబడి/ఉద్యోగం నుండి తొలగించారు

11= Other (please specify):
ఇతరములు(దయచేసి తెలియచేయండి):_______________________________________

9 **Do you receive any of the following benefits**

మీరు ఈ క్రింది ప్రయోజనాలు ఏవైనా పొందారా?

	Yes అవును	No లేదు	If yes, run by అయితే ఎవరి ద్వారా నడుస్తుంది	
			ప్రభుత్వ Govt	ప్రభుత్వేతర NGO
A. Social security grant<br>సాంఘిక భద్రతా మంజూరు (గ్రాంటు)	1	2	1	2
B. Disability grant<br>అంగవైకల్య మంజూరు	1	2	1	2
C. Pension<br>భరణము(పెన్షన్)	1	2	1	2
D. .Family Allowance<br>కుటుంబ భత్యము	1	2	1	2
E. Other (specify)<br>ఇతరములు (తెలియచేయండి)___________________	1	2	1	2

10 Are you involved in any of the following

మీరు ఈ క్రింది వాటిలో దేనిలోనైనా పాల్గొన్నారా?

	Yes అవును	No లేదు	If yes, run by అయితే ఎవరి ద్వారా నడుస్తుంది	
			ప్రభుత్వ Govt	ప్రభుత్వేతర NGO
A. Self Help Groups<br>స్వయం సహాయక బృందం	1	2	1	2
B. Microfinance Groups<br>సూక్ష్మ ఋణాల బృందం	1	2	1	2
C.<br>పనికి నగదు పథకం	1	2	1	2
D. Others (Specify)<br>ఇతరములు (తెలియచేయండి) -----------	1	2	1	2

F. HEALTH AND ANTENATAL CARE
ఆరోగ్యము మరియు జన్మసంబంధ సంరక్షణ

These questions are about your health

ఈ ప్రశ్నలు మీ ఆరోగ్యము గురించినవి

F.1. CASES ONLY (all ages)

కేసులు మాత్రమే (అన్ని వయసులవారికి)

Note to Interviewer: If participant screened positive via self report say:

ఇంటర్వ్యూ చేసే వారికి గమనిక : ఒకవేళ స్వీయనివేదిక ద్వారా అభ్యర్థి పరీక్షలో సానుకూలమని గుర్తిస్తే చెప్పండి:

Your responses to our earlier questions and examinations indicate that you may have difficulties in certain areas related to your health.

మునపటి ప్రశ్నలకు మరియు పరీక్షలకు మీ ప్రతిస్పందన ద్వారా మీకు మీ ఆరోగ్యసంబంధిత కొన్ని విషయాలలో సమస్యలున్నాయని సూచిస్తున్నది.

1. What do you think is the cause of the difficulties you face in your health? (tick all that apply)

   మీ ఆరోగ్య విషయంలో మీరు ఎదుర్కొంటున్న సమస్యలకు కారణం ఏమనుకొంటున్నారు? (సరియైన వాటినన్నిటిని టిక్ చెయ్యండి)

1 = From Birth
పుట్టుకతో

2 = Trauma
గాయం

3 = Illness
అనారోగ్యం

4 = Aging
వయోభారం

5 = Other
ఇతరాలు

2 How old were you when it started? 00 = from birth ☐ Years

మీకు ఎంత వయసువుండగా ఇది మొదలయ్యింది? పుట్టుకతో సంవత్సరాలు

99 = Don't know/refused
తెలియదు/తిరస్కరించబడింది

F2. All CASES AND CONTROLS

అన్ని కేసులు/నియంత్రణలు

1. Have you ever been diagnosed by a medical doctor with any of the following?

మీకు ఎప్పుడైనా వైద్యుడు చేసిన పరిక్షల ద్వారా క్రింది వాటిలో ఏమైనా నిర్ధారించడం జరిగిందా.

	Yes అవును	No కాదు		Yes అవును	No కాదు
**1= High Blood Pressure** అధిక రక్తపోటు	1	2	9= COPD దీర్ఘకాలిక వ్యాదులు	1	2
2= Diabetes మధుమేహం	1	2	**10= Emphysema ఎంఫిసెమా**	1	2
3= Arthritis కీళ్ళవ్యాధి	1	2	11= HIV హెచ్.ఐ.వి	1	2
4 =Heart Disease గుండె జబ్బులు	1	2	12= Cancer క్యాన్సర్	1	2
5= Asthma, asthmatic bronchitis or allergic bronchitis **ఆస్తమా, ఉబ్బస శ్వాస నాళముల వాపు లేదా అలెర్జీ శ్వాస నాళముల వాపు**	1	2	If yes, what type of cancer అవును అయితే, ఎలాంటి రకమైన క్యాన్సర్__________________	1	2
6= Stroke (paralytic attack) గుండె నొప్పులు	1	2	13= Any other health condition? ఇతర ఏమైనా ఆరోగ్య పరిస్థితి	1	2
7= Thyroid problem థైరాయిడ్ సమస్యలు	1	2	If yes, specify ______________________ అవును అయితే, తెలియజేయండి	1	2
8=Tuberculosis టి.బి.	1	2			

2 Have you had any serious health problems during the last twelve months, including but not limited to those you may have mentioned above?

గత 12 నెలలుగా మీకు ఏవయినా తీవ్రమైన ఆరోగ్య సమస్యలు కలిగాయా? పైన పేర్కొన్నవి కాకుండా (వర్తించే వాటినన్నింటిని గుర్తించండి)

0 = No If NO, GO TO SECTION F3

0 = లేదు ,   లేదు అయినచో తరువాత విభాగానికి **వెళ్ళండి**

1 = Yes   అవును

3 If yes, what type of serious health event(s) or problem(s) did you experience during this period? (tick all that apply)

ఒకవేళ 'అవును' అయితే ఏ రకమైన తీవ్ర ఆరోగ్య పరిస్థితులు/సమస్య(లు) మీరు ఈ సమయంలో అనుభవించారు?

(సరియైన వాటినన్నిటిని టిక్ చేయండి)

1= Severe Diarrhea (with dehydration or for more than 14 days)
తీవ్రమైన అతిసారవ్యాది (14 రోజులకన్నా ఎక్కువ నిర్జలీకరణముతో)

2= Acute respiratory tract infection/pneumonia
తీవ్రమైన శ్వాసనాళ సంక్రమణవ్యాధి/ న్యుమోనియా

3= Malaria
మలేరియా

4= Eye Infection/eye problems
కంటికి సంక్రమించువ్యాధి/కంటి సమస్య

5= Ear infection/ear or hearing problems
చెవికి సంక్రమణవ్యాధి/చెవి లేదా వినికిడి సమస్య

6= Malnutrition
పోషకాహారలోపం

7= Vaccine-preventable disease (*including measles, chickenpox, mumps, rubella, tetanus, TB, whopping cough)*
వాక్సిన్ తో నివారించగల వ్యాధి(గదవబిళ్లలు,రుబెల్లా, ధనుర్వాతము, క్షయ(టిబి),కోరింతదగ్గు తట్లు,ఆట్లమ్మలతో కలిపి)

8= Chronic Illness
దీర్ఘకాల అనారోగ్యము

9= Accident/Injuries
దుర్ఘటనలు/దెబ్బలు

10= Jaundice

పసిరికలు

11= Skin Diseases

చర్మ వ్యాదులు

12= Don't know/ no information provided

తెలియదు/సమాచారం ఏది ఇవ్వలేదు

13= Other, specify

ఇతరములు, తెలియజేయండి______________________

4 Where did you seek advice or treatment?

మీరు ఎక్కడ సలహా లేదా చికిత్సని తీసుకుంటున్నారు?

1 = did not seek advice or treatment **If Response = 1, GO to Q5**

ఎక్కడ సలహా లేదా చికిత్సను తిసుకోలేకపోతే . ఒకవేళ ప్రతిస్పందన = 1 అయితే Q5 కి వెళ్ళండి

2 = village/community health worker or agent
గ్రామ/సంఘ ఆరోగ్య కార్యకర్త లేదా ప్రతినిధి

3= hospital
ఆసుపత్రి

4= pharmacy
ఔషధశాల (మందుల దుకాణము)

5= mobile clinic
మొబైల్ క్లినిక్

6= private doctor

ప్రైవేటు వైద్యుడు

7= health centre/post (including RMPs)
ఆరోగ్య కేంద్రం/ పోస్ట్ (RMP డాక్టర్లను కలిపి)

8= traditional healer
సాంప్రదాయిక వైద్యుడు

9= other, specify
ఇతరములు, తెలియజేయండి_________________________________

(Responses 2–9:) All other responses go to Section F3

ఇతర అన్ని సమాధానాలకు సెక్షన్ 3 కి వెళ్ళండి

5. If you did not seek advice or treatment, what was the reason? (3 answers possible)
ఒకవేళ మీరు సలహాని లేదా చికిత్సని తీసుకోకపోయినచో, దానికిగల కారణములు ఏమిటి?

(మూడు సమాధానములు వరకు సాధ్యము)

Financial difficulties
ఆర్ధిక ఇబ్బందులు:

1 = I was refused because I had no money (or not enough)
డబ్బులేని(లేదా తగినంత డబ్బులేని) కారణము చేత నేను తిరస్కరించబడ్డాను,

2 = I had difficulty to get food for myself during my stay
నేను అక్కడ వున్న సమయంలో నా భోజనానికి ఇబ్బంది కలిగింది

3 = I didn't have money to pay for treatment
చికిత్సకోసం చెప్పించటానికి నావద్ద డబ్బులేదు

4 = I didn't have money for medication/objects
నావద్ద మందులకు/అవసరమైన వస్తువులకు డబ్బు లేదు

Transport, access difficulties
రవాణా,అందుబాటు ఇబ్బందులు.

5= there was no available transportation/it's very far away

రవాణా సదుపాయము లేదు/అది చాలా దూరము

6 = I had difficulty to find the money for transportation

ప్రయాణ ఖర్చులకు డబ్బు సమకూర్చుకోవటం కష్టం అయ్యింది

7= No transport - refused travel on public transport

రవాణా సౌకర్యం లేదు/ ప్రజారవాణాలో తిరస్కరించబడ్డాను

8 = I had difficulty to find someone to go with me because nobody had time to take me

తోడుగా వచ్చేవారిని సంపాదించలేకపోవుటచేత/నన్ను తీసుకెళ్లటానికి ఎవరికి ఖ

9= I did not ask anybody because I felt that it was a waste of time
నేను ఎవరిని అడగాలన్నా సమయం వృధాచేసుకోవటమే అనిపించి ఎవరినీ అడగలేదు

Difficulties at the health service
ఆరోగ్యసేవా కేంద్రాల వద్ద ఇబ్బందులు:

10=I did not have the documents required to access health services
ఆరోగ్యసేవాలు పొందటానికి కావలసిన పత్రాలు నాదగ్గర లేవు

11= there was no available medication
అందుబాటులో మందులు లేకపోవటం

12 = there was no service available for my need (condition)
నా అవసరానికి(పరిస్థితికి) పనికొచ్చే సేవలు అందుబాటులో లేవు

13 = I was refused because I am disabled

నా వైకల్యము దృష్ట్యా తిరస్కరించబడ్డాను.

14= attitude of medical staff was negative

వైద్యసిబ్బంది యొక్క వైఖరి ప్రతికూలంగా ఉండెను

15= The equipment that they gave is not very useful
వారు ఇచ్చిన పరికరాలు అటంత ఉపయోగంగా లేవు

16 = there is no female professional

అక్కడ మహిళా నిపుణురాలు లేరు

17 = Family members did not agree with me seeking treatment

చికిత్స తీసుకోవటానికి కుటుంబ సభ్యులు అంగీకరించలేదు

18 = no difficulty

ఇబ్బంది లేదు

19=other, specify ___________________________

ఇతరములు, తెలియచేయండి....

F3. REPRODUCTIVE HEALTH: MARRIED, SEPARATED, DIVORCED OR WIDOWED FEMALE CASES AND CONTROLS AGED 15 to 49 ONLY. ALL OTHER PARTICIPANTS GO TO SECTION F4
సంతానోత్పత్తి ఆరోగ్యం:పెళ్లి అయిన , విడిగా ఉంటున్న , విడాకులు తీసుకున్న లేదా విధవరాలు గా మహిళలు మరియు కంట్రోల్స్ వయసు 15 నుంచి 45 మాత్రమే. (ఇతరులు సెక్షన్ F4 కి వెళ్ళండి)

1 Do you have any children? 0 = No **If NO, go to Q3**

మీరు పిల్లలు కలిగి ఉన్నారా? 0 = లేదు , లేదు అయినచో Q3 **వెళ్ళండి**

1 = Yes అవును

2 How many children do you have today (excluding those who have died)?

ఈ రోజుకి మీకు ఎంతమంది పిల్లలు ఉన్నారు?(చనిపోయిన వారిని మినహాయించి)

3 Did you have any pregnancies that ended before term (i.e. Still birth, miscarriage or abortion)?

ఇంతకు ముందు కాలానికి ముందే కోల్పోయిన గర్భధారణలు ఏవైనా ఉన్నాయా? (అనగా పుట్టిన వెంటనే చనిపోవటం, గర్భస్రావాలు వంటివి)

0 = No **If NO, GO TO NEXT SECTION F4**

0 = లేదు , లేదు, అయినచో తరువాత విభాగమునకు **వెళ్ళండి**

1 = Yes అవును

4 If yes, how many pregnancies ended before term

అవును అయినచో, ఎన్ని గర్భాలు అలా విచ్ఛిన్నమయ్యాయి?

F4. PREGNANCY CARE: WOMEN WITH CHILDREN UNDER 5 ONLY. ALL OTHER PARTICIPANTS GO TO SECTION F5

గర్భసంరక్షణ : 5సంవత్సరాల వయసు లోబడిన పిల్లలు కలిగియున్న మహిళలు(**ఇతరులు సెక్షన్ F4 కి వెళ్ళండి**)

5. Are any of your children aged 5 or younger? 0 = No కాదు **If NO, GO TO NEXT SECTION**

మీ పిల్లలలో ఎవరైనా 5 సంవత్సరాలు లేదా తక్కువ వయసు గలవారు ఉన్నారా? **1 = Yes** **అవును** **కాదు అయితే , తరువాతి సెక్షన్ కి వెళ్ళండి**

I would now like to ask you some questions about your children born in the last 5 years. Please answer questions about the last child born in this period

గత 5సంవత్సరాలలో మీకు పుట్టిన పిల్లలగురించి కొన్ని ప్రశ్నలు అడగాలనుకొంటున్నాను. **ఈ కాలవ్యవధిలో పుట్టిన ఆఖరి పాప/బాబు గురించిన ప్రశ్నలకు దయచేసి సమాధానములు ఇవ్వండి.**

Note to Interviewer: If no children born in the last five years, go to Section F5

ఇంటర్వ్యూ చేసే వారికి గమనిక : గత ఐదు ఏళ్ళలో పిల్లలు ఎవరు పుట్టని యెడల తదుపరి విభాగమునకు వెళ్లండి

6 Did you see anyone for antenatal care during this time?

ఈ కాలంలో గర్భసంరక్షణ కోసం ఎవరినైనా సంప్రదించారా?

0 = No **If NO, Go to Q7**

0 = లేదు లేదు అయినచో, Q7 కు **వెళ్ళండి**

1 = Yes అవును

7 Whom did you see? Anyone else?

ఎవరిని కలిసారు? ఇతరులు ఎవరైనా?

1 = Health personnel/Doctor
ఆరోగ్యసిబ్బంది/ వైద్యులు

2 = Nurse/midwife
నర్సు/ మంత్రసాని

3 = Auxiliary Midwife
సహాయకమంత్రసాని

4 = Traditional Birth Attendant
సాంప్రదాయ జనన పరిచారిక

5 = Community/village health worker
సంఘు/గ్రామ ఆరోగ్య కార్యకర్త

6 = Other (please specify)
ఇతరములు, తెలియజేయండి______________________

8 Where did you give birth to [name]?

*Probe to identify source. If unable to determine if public/private sector write the name of the place*__________________

(పేరు) యొక్క కాన్పు ఎక్కడ జరిగింది? ఏ రకమైన వ్యక్తి అని గుర్తించటానికి దర్యాప్తు చేసి పేర్కొన్న విషయాన్ని నమోదు చెయ్యండి. ప్రదేశాన్ని గుర్తించటానికి దర్యాప్తు చెయ్యండి. ప్రభుత్వ రంగం/ ప్రైవేటు రంగం నిర్ణయించలేని పక్షంలో ఆ ప్రదేశముయొక్క పేరు వ్రాయండి

1 = Home (Your home)
ఇల్లు(మీ ఇల్లు)

2 = other home

ఇతరుల ఇల్లు

3= Public sector Govt. hospital

ప్రభుత్వ రంగ ఆసుపత్రి

4 = Public sector Govt. health centre
ప్రభుత్వ రంగ ఆరోగ్యకేంద్రము

5 =Public sector Govt health post

ప్రభుత్వ రంగ ఆరోగ్యపోస్ట్ లు

6 = Other public sector (specify)

ఇతర ప్రభుత్వ రంగం, తెలియచేయండి ____________________

7 = Private medical sector/private hospital clinic
ప్రైవేటు వైద్యరంగం/ ప్రైవేటు ఆసుపత్రి

8 = Dispensary
వైద్య చికిత్సాకేంద్రం

9 = Other private medical sector (specify)

ఇతర ప్రైవేటు వైద్య రంగం, తెలియచేయండి __________________________

10 = Other (specify)

ఇతరములు, తెలియచేయండి____________

9 Who assisted with the delivery of [Name]? Anyone else?

Probe for the types of person and record all mentioned. If respondent says no one assisted, probeto determine whether any adults were present at delivery

(పేరు) కాన్పు సమయంలో ఎవరు సహాయము చేసారు? ఇతరులెవరైనా?

ఏ రకం వ్యక్తులు వున్నారో దర్యాప్తు చేసి తెలియచేసిన విషయాన్ని నమోదు చెయ్యండి. ఒకవేళ ప్రతివాది ఎవరు లేరు అని చెప్పినట్లయితే కాన్పు సమయంలో ఎవరైనా పెద్దలు వున్నారా అనే విషయాన్ని దర్యాప్తుచేసి గుర్తించండి.

1 = Doctor

ఆరోగ్యసిబ్బంది/ వైద్యులు

2 = Nurse/Midwife

నర్స్/మంత్రసాని

3 = Auxiliary Midwife

సహాయ మంత్రసాని

4 = Traditional birth attendant

సాంప్రదాయంగా కాన్పులు చేసేవారు

5 = Relative/friend

బంధువులు/ స్నేహితులు

6 = Other (specify)

ఇతరములు, (తెలియజేయండి____________________________)

7 = No one assisted

ఎవరు సహాయపడలేదు

9. Did [name] ever have any vaccinations to prevent him/her getting diseases, including vaccinations received in a national

immunization coverage days

(పేరు) కు వ్యాది వచ్చినా తరువాత నివారించడానికి పాప/బాబు కి ఎప్పుడైనా టీకాలు వేయించారా, జాతీయ రోగనిరోధక దినాలలో ఇచ్చే టీకాలు

కలుపుకొని

0 = No లేదు

1 = Yes అవును

G **REHABILITATION: CASES ONLY - CONTROLS GO TO SECTION I**
పునరావాస కేసులు మాత్రమే

I am now going to ask you some questions about some services specifically for people with disabilities that you may or may not have heard of or have used now or in the past<br>ఇప్పుడు నేను ప్రత్యేకంగా వైకల్యం ఉన్నవారికోసం కొన్ని సేవలగురించి ప్రశ్నలు అడగబోతున్నాను,<br>అది మీరు గతంలో విని లేదా ఉపయోగించి ఉండవచ్చు లేదా ఉండకపోవచ్చును.		1.1 Have you ever heard of this type of service?<br>మీరు ఎప్పుడైనా ఈ రకం సేవలగురించి విన్నారా?	1.2 Have you ever needed this service?<br>మీకు ఎప్పుడైనా ఈ సేవల అవసరం పడిందా?	1.3 Have you ever received this service?<br>మీరు ఎప్పుడైనా ఈ సేవలు పొందారా?	1.4 If yes, are you currently receiving or using it?<br>**అవును అయితే ప్రస్తుతం మీరు తీసుకుంటున్నారా లేదా వాడుతున్నారా?**	1.5 If reported needing (Yes to Q1.2) but not receiving a service (No to Q1.3), ask why have you not received it?<br>ఒకవేళ అవసరం ఉందని నివేదించినా (ప్ర.1.2కి అవును)కాని సేవను అందుకోకుంటే(ప్ర.1.3కి కాదు)ఎందుకు పొందటలేదు అని అడుగు?	1.6 If reported once receiving/using service (Yes to Q1.3) but not receiving it now (No to Q1.4), ask why are you no longer receiving it?<br>ఒకవేళ ఒకప్పుడు పొందేవారు/సేవలు ఉపయోగించేవారు(ప్ర.1.3కి అవును)కాని ఇప్పుడు పొందుటలేదు(ప్ర.1.4కి లేదు) మీరు ఎందుకు అది ఇకపై స్వీకరించుటలేదు?
		0 = No<br>0 = లేదు<br>(go to next service)<br>(తరువాత సేవకు వెళ్లు)<br>1 = Yes<br>1 = అవును	0 = No<br>0 = లేదు<br>(go to next service)<br>(తరువాత సేవకు వెళ్లు)<br>1 = Yes<br>1 = అవును	0 = No<br>0 = లేదు<br>→ Q 1.5<br>ప్ర 1.5<br>1= Yes<br>1 = అవును<br>→ Q 1.4<br>ప్ర.1.4	0 = No<br>0 = లేదు<br>→ Q 1.6<br>ప్ర 1.6<br>1= Yes<br>1 = అవును<br>(తరువాత సేవకు వెళ్లు)	1 = Too expensive<br>చాలా ఖరీదు<br>2 = Too far/no transport<br>చాలా దూరం/రవాణా లేదు<br>3 = Discriminating<br>వివక్షత<br>4 = Communication barriers<br>సంభాషణా ఆటంకాలు<br>5 = Don't know where to access<br>ఎక్కడ దొరకుతుందో తెలియదు<br>6 = Service not available<br>సేవ అందుబాటులో లేదు<br>7= Other (specify)_______<br>ఇతరములు, తెలియజేయండి<br>_________________________<br>**మూడు ప్రతిస్పందనల వరకు అనుమతించబడతాయి.**	1 = Too expensive<br>చాలా ఖరీదు<br>2 = Too far/no transport<br>చాలా దూరం/రవాణా లేదు<br>3 = Not longer available<br>ఇప్పుడు అందుబాటులో లేదు<br>4 Communication/ language barriers<br>సంభాషణా/భాషా ఆటంకాలు<br>5 = Don't know where to access<br>ఎక్కడ దొరకుతుందో తెలియదు<br>6 = Not really helping me<br>నాకు అంత ఉపయోగపడుట లేదు<br>7 = Not satisfied with services<br>సేవలతో సంతృప్తి కలగ లేదు<br>8= No longer need the service<br>ఇకపై సేవలు అవసరం లేదు<br>9=Broken and unable to repair<br>విరిగినది మరియు బాగు సాధ్యము కాలేదు<br>**మూడు ప్రతిస్పందనల వరకు అనుమతించబడతాయి.**
a	**Medical rehabilitation** (e.g. physiotherapy, occupational therapy, speech and hearing therapy etc)<br>**వైద్య పునరావాసం** (ఉదా.ఫిజియోథెరపీ,ఆక్యుపేషనల్ థెరపీ, ప్రసంగ మరియు వినికిడి చికిత్స మొ..)	0 1	0 1	0 1	0 1		
b	**Assistive devices service** (e.g. Sign language interpreter, wheelchair, hearing/visual aids, Braille etc.)	0 1	0 1	0 1	0 1		

	**ఉపకరణ పరికరాల సేవ** (ఉదా.సంకేతభాషా అనువాది,చక్రాలకుర్చీ,దృశ్య/శ్రవణ ఉపకరణాలు,బ్రెయిలీ మొ..)						
c	**Specialist educational services (e.g. therapist, school support services)**<br>**ప్రత్యేక విద్యా సేవలు**(ఉదా.చికిత్సకుడు, పాఠశాల మద్దతు సేవలు) వృత్తి శిక్షణ (ఉపాధి నైపుణ్య శిక్షణ మొ..)	0 1	0 1	0 1	0 1		
d	**Vocational Training** (e.g. Employment skills training, etc.)<br>**ప్రత్యేక విద్యా సేవలు**(ఉదా.చికిత్సకుడు, పాఠశాల మద్దతు సేవలు)<br>వృత్తి శిక్షణ (ఉపాధి నైపుణ్య శిక్షణ మొ||)	0 1	0 1	0 1	0 1		
e	**Counselling for person with a disabiliy (e.g. Physchologist, psychiatrist, counsellor)**<br>**వైకల్యపు వ్యక్తికి కౌన్సిలింగ్** (ఉదా.శరీరధర్మ శాస్త్రవేత్త, మానశిక వైద్యుడు,కౌన్సలర్)	0 1	0 1	0 1	0 1		
f	**Counselling for parent/family**<br>**తల్లిదండ్రులకు/కుటుంబ సభ్యులకు కౌన్సిలింగు**	0 1	0 1	0 1	0 1		
g	**Welfare services** (e.g. social worker, disability grant, etc)<br>**సంక్షేమ సేవలు (ఉదా. సామాజిక కార్యకర్తలు, వైకల్య గ్రాంటు మొ..)**	0 1	0 1	0 1	0 1		
h	**Health services** (e.g. at a primary health care clinic, hospital, home health care services etc.)<br>**ఆరోగ్యసేవలు** (ఉదా.ప్రాథమిక ఆరోగ్య చికిత్సాకేంద్రం,ఆసుపత్రి, కుటుంబ ఆరోగ్య సంరక్షణా సేవలు మొ..)	0 1	0 1	0 1	0 1		
i	**Health information** (e.g. From the radio, tv, at schools, clinics, hospital etc.)<br>**ఆరోగ్య సమాచారం** (ఉదా. రేడియో, టివి, పాఠశల వద్ద, చికిత్సాకేంద్రం, ఆసుపత్రులు మొ..)	0 1	0 1	0 1	0 1		
j	**Traditional healer/faith healer**<br>**సాంప్రదాయిక హీలర్, విశ్వాస హీలర్**	0 1	0 1	0 1	0 1		
k	**Legal advice related to having a disability**<br>**వైకల్యానికి సంబంధించిన న్యాయ సలహా**	0 1	0 1	0 1	0 1		
l	**Specialist health services** (e.g. Surgery, ear/eye medical, psychiatry services)<br>**ప్రత్యేక ఆరోగ్య సేవలు** (శస్త్రచికిత్స, చెవి/కంటి వైద్యం, మనోరోగ చికిత్సలు)	0 1	0 1	0 1	0 1		

H. ASSISTIVE DEVICES: CASES ONLY - CONTROLS GO TO SECTION I
సహాయక పరికరాలు:కేసులు మాత్రమే – కంట్రోల్స్ అయితే సెక్షన్ ఐ కి వెళ్ళండి

Note to Interviewer: Read list of devices that are relevant to difficulty categories of impairment
ఇంటర్వ్యూ చేసే వారికి గమనిక :కష్టమైన వైకల్య వర్గాలకు సంబంధించిన పరికరాల జాబితాను చదవండి

		1.1 I am going to read you a list of assistive devices. For each please tell me if you use it, need it but don't use it, or don't need it<br>నేను మీకు సహాయక పరికరాలయొక్క జబితాను చదువబోతున్నాను. ఒక్కొక దానిని వాడుతున్నారా, అవసరం ఉంది కానీ వాడుటలేదు,లేదా అవసరం లేదు అని దయచేసి చెప్పండి.	1.2 If used, is it in good working order?<br>ఒకవేళ వాడుంటే,పనిచేసే స్థితిలో ఉందా?	1.3 If used, where did you get the assistive device?<br>ఒకవేళ వాడుంటే,ఉపకరణ పరికరం ఎక్కడ నుండి పొందారు?	1.4 If reported, needing but not using: what is the main reason why don't you use it?<br>ఒకవేళ అవసరం ఉంది కాని వాడుట లేదు అని నివేదించి ఉంటే,ముఖ్యకారణం ఏమిటి, ఎందుకు వాడుట లేదు?
Difficulty Category<br>సమస్య రకం	పరికరం	1 = Use it ఇది ఉపయోగించండి<br>2 = Need it, but don't use it -> Q 1.4 ఇది అవసరం, కానీ వాడుట లేదు ప్ర.>1.4<br>3 = Don't need/NA -> next device<br>అవసరం లేదు/వర్తించదు -> తరువాత పరికరం<br>4 = Don't know what it is -> Next device<br>అది ఏమిటో తెలియదు>తరువాత పరికరం	1 = Yes అవును<br>0 = No లేదు<br>3 = N/A<br>వర్తించదు	1 = Private provider ప్రైవేటు ప్రదాత తరువాత<br>2 = Government health service ప్రభుత్వ ఆరోగ్య సేవ<br>3 = Government service (not health) Next ప్రభుత్వ సేవ(ఆరోగ్యం కాదు) పరికరం<br>4 = NGO<br>ప్రభుత్వేతర సంస్థ<br>5 = Friend/relative<br>స్నేహితులు/బంధువులు<br>6 = Other 7 = Don't know<br>ఇతరములు 7 = తెలియదు	1 = Not really helping me నిజానికి అంత ఉపయోగపడటంలేదు<br>2 = Not satisfied with device<br>పరికరం సంతృప్తికరంగా లేదు<br>3= No longer need the device<br>పరికరం ఇంక అవసరం లేదు<br>4=Broken and unable to repair (cost)<br>చెడిపోయింది,బాగుచేయలేకపోతున్నాము(ఖర్చు)<br>5=Broken and unable to repair (too far)<br>చెడిపోయింది,బాగుచేయలేకపోతున్నాము (చాలా దూరం)<br>6= Broken and unable to repair (not available)<br>చెడిపోయింది,బాగుచేయలేకపోతున్నాము (అందుబాటులో లేదు)
Seeing<br>చూచుట	a.Eye Glasses<br>కళ్లద్దాలు				
	b.Magnifying glass<br>భూతద్దం				
	c. Telescoping Lenses/glasses<br>దూరదర్శని కటకాలు/ అద్దాలు				

	d. Enlarge print పెద్దది చేయు ముద్రణ				
	e. Braile బ్రెయిలీ				
	f. Other, specify ఇతరములు, తెలియచేయండి.....				
Hearing వినికిడి	g. Hearing Aid శ్రవణోపకరణాలు				
	h. Computer కంప్యూటర్				
Mobility కదలిక	i.Wheel chair చక్రాల కుర్చీ				
	j. Crutches ఊతకర్రలు				
	k. Walking stick చేతికర్ర				
	l. White cane వైట్ కేన్				
	m. Guide గైడ్				
	n. Standing Frame స్టాండిగ్ ఫ్రేము				
	o. o. Other, specify ఇతరములు, తెలియజేయండి....				

2. Do you use any other assistive devices
మీరు ఏవైనా ఇతర సహాయ పరికరాలను
వాడుతున్నారా?

0 = No
లేదు
1 = Yes
అవును

If NO, Go to Q4 ప్రశ్న 4 కు **వెళ్ళండి**
If YES, Go to Q3 ప్రశ్న 3కు **వెళ్ళండి**

3.If yes, please tell me what they are:
(use device list below)
సమాధానము అవును అయినచో, అది ఏమిటో చెప్పండి :
(క్రిందనున్న పరికరముల పట్టికను ఉపయోగించండి)

కోడ్:

if other specify
ఇతరములు, అయితే తెలియజేయండి ______________________________

4. Are there any assistive devices you think you need but do not have?
మీరు కావాలి అనుకొంటున్న కాని మీకు లేని సహాయక పరికరాలు ఏవైనా ఉన్నాయా?

0 = No
లేదు
1 = Yes
అవును

If NO, go to SECTION I
కాదు అయితే సెక్షన్ ఐ కి **వెళ్ళండి**
If YES, Go to Q 5
అవును అయితే , ప్రశ్న 5కు **వెళ్ళండి**

5. If yes, please tell me what they are: (use device list below)
సమాధానము అవును అయినచో, అది ఏమిటో చెప్పండి (క్రిందనున్న పరికరముల పట్టికను ఉపయోగించండి)

కోడ్:

if other specify
ఇతరములు, అయితే తెలియజేయండి ______________________

Eyeglasses = 1<br>కంటి అద్దాలు = 1	Walking Stick = 4<br>చేతికర్ర = 4	Walking Frame = 7<br>వాకింగు ఫ్రేము= 7	Amplified Telephone = 10<br>ఆంప్లిఫైడ్ టెలిఫోన్ = 10	Computers and/or special computer software = 13<br>కంప్యూటర్ లు మరియు ప్రత్యేక కంప్యూటర్ సాఫ్ట్ వేర్ =13
Hearing Aid = 2<br>శ్రవణోపకరణం = 2	White cane = 5<br>వైట్ కేను = 5	Communication Board = 8<br>కమ్యూనికేషన్ ఆన్ బోర్డ్= 8	Toilet Seat Raiser = 11<br>టాయిలెట్ సీట్ రైజరు = 11	Others (specify) = 14<br>ఇతరములు,(తెలియజేయండి)=14
Wheelchair = 3<br>చక్రాలకుర్చీ = 3	Crutches = 6<br>ఊతకర్రలు = 6	Braille = 9<br>బ్రెయిలీ=9	Bath and shower seats = 12 బాత్<br>మరియు షవర్ సీట్ లు 12	

II. ACTIVITY LIMITATIONS AND PARTICIPATION RESTRICTIONS: ALL CASES AND CONTROLS
కార్యాచరణా మరియు పాల్గొనుటలో పరిమితులు(అన్ని కేసులకు నియంత్రణలకు)

ACTIVITY LIMITATION
కార్యాచరణ పరిమితులు

I would like to know how difficult it is for you to perform this activity WITHOUT any kind of assistance at all?
(Without the use of assistive devices - either technical or personal)
ఎటువంటి సహాయము లేకుండా ఈ పనులు చేయుట మీకు ఎంత కష్టమో నేను తెలుసుకోవాలనుకొంటున్నాను

(సహాయోపకరణాలు వాడకుండా - సాంకేతికంగా కానీ లేదా వ్యక్తిగతంగా)

A		No difficulty కష్టం లేదు	Moderate difficulty భరించగలిగే కష్టం	Severe difficulty చాలా కష్టం	Unable to do చెయ్యలేకపోతున్నా	తెలియదు
	watching/looking/seeing తిలకించుట/వీక్షించుట/చూచుట	1	2	3	4	5
	listening/hearing శ్రవణం/ వినుట	1	2	3	4	5
	learning to read/write/count/calculate చదువుట/వ్రాయుట/లెక్కించుట/గణించుటలను నేర్చుకొనుట	1	2	3	4	5
	acquiring skills (manipulating tools, painting, carving etc.) నైపుణ్యాన్ని సంపాదించు(పనిముట్ల వాడుక,చిత్రలేఖనం,శిల్పకళ మొ..)	1	2	3	4	5
	thinking/concentrating ఆలోచన/ఏకాగ్రత	1	2	3	4	5
	reading/writing/counting/calculating చదువుట/వ్రాయుట/లెక్కించుట/గణించుట	1	2	3	4	5
	solving problems సమస్యా పరిష్కారం సమస్యా పరిష్కారం	1	2	3	4	5
	understanding others (spoken, written or sign language) ఇతరులను అర్థంచేసుకొనుట(మాట్లాడుట,వ్రాయుట,లేదా సైగలుబాష)	1	2	3	4	5
	producing messages (spoken, written or sign language) సమాచారోత్పత్తి (మాట్లాడుట,వ్రాయుట,లేదా సైగలుబాష)	1	2	3	4	5
	communicating directly with others ప్రత్యక్షంగా ఇతరులతో సంభాషించుట	1	2	3	4	5
	staying in one body position నిటారుగా నిలబడుట	1	2	3	4	5
	changing a body position (sitting/standing/bending/lying) శరీర స్థితి మార్పులు (కూర్చునుట, నిలబడుట, వంగుట, పడుకొనుట)	1	2	3	4	5
	transferring oneself (moving from one surface to another) స్వయంగా కదలుట(ఒక ప్రదేశం నుండి వేరొక ప్రదేశానికి)	1	2	3	4	5
	lifting/carrying/moving/handling objects వస్తువులను ఎత్తుట/మోయుట/కదపుట/వాడుట	1	2	3	4	5
	fine hand use (picking up/grasping/manipulating/releasing) సున్నితమైన వాడుక (తీసుకొనుట/గ్రహించు/హస్తలాఘువం/వదిలిపెట్టడం)	1	2	3	4	5
	hand & arm use (pulling/pushing/reaching/throwing/catching) చేతులు మరియు మో చేయి వాడుక(లాగుట/త్రోయుట/చేరుట/విసరుట/అందుకొనుట)	1	2	3	4	5
	Walking నడచుట	1	2	3	4	5
	moving around (crawling/climbing/running/jumping) అటునిటు తిరుగుట(ప్రాకుట/ఎక్కుట/పరిగిడుట/దుముకుట)	1	2	3	4	5

PARTICIPATION RESTRICTION   పాల్గొనుటలో పరిమితులు

2 Do you have any difficulty performing this activity in your current environment? Now I would like to know whether you have difficulties even with the help of assistive devices or another person
మీకు ప్రస్తుత వాతావరణంలో ఈ చర్యలు చేయటంలో ఏవైన ఇబ్బందులు ఉన్నాయా? ఇప్పుడు మీకు సహాయక పరికరాలు మరియు వ్యక్తుల సహాయంతో కూడా ఇబ్బందులు ఉన్నాయా, అని తెలుసుకోవాలి అనుకొనుచున్నాను.
(ప్రస్తుత వాతావరణం అనగా మీరు ఎక్కువ సమయం ఉండే, పనిచేసే, ఆడుకొనే ప్రదేశం).

	No difficulty కష్టం లేదు	Moderate difficulty భరించగలిగే కష్టం	Severe difficulty చాలా కష్టం	Unable to do చెయ్యలేక పోతున్నా	Don't know తెలియదు
washing oneself స్వయంగా శుభ్రపరచుకోగలగటం	1	2	3	4	5
care of body parts, teeth, nails and hair శరీర భాగాల, దంతముల,గోళ్లు మరియు జుట్టు యొక్క జాగ్రత్త	1	2	3	4	5
Toileting కాలకృత్యాలు	1	2	3	4	5
dressing and undressing దుస్తులు ధరించటం మరియు తీసివేయటం	1	2	3	4	5
eating and drinking తినటం మరియు త్రాగటం	1	2	3	4	5
shopping (getting goods and services) కొనుగోలు (సరుకులు మరియు సేవలు పొందటం) 8 పైవారికి మాత్రమే	1	2	3	4	5
preparing meals (cooking) భోజనం తయారు చేయడం(వంట)8 పైవారికి మాత్రమే	1	2	3	4	5
doing housework (washing/cleaning) ఇంటి పనులు చేయుట (బట్టలు ఉతకటం, శుభ్రంచేయటం)8 పైవారికి మాత్రమే	1	2	3	4	5
taking care of personal objects (mending/repairing) సొంత వస్తువుల జాగ్రత్త(బాగుచేసుకోవటం)18 పైవారికి మాత్రమే	1	2	3	4	5
taking care of others ఇతరుల జాగ్రత్త తీసుకోవటం	1	2	3	4	5
making friends and maintaining friendships స్నేహం చెయ్యటం, స్నేహం నిలబెట్టుకోవటం	1	2	3	4	5
interacting with persons in authority (officials, village chiefs) ఇతర వ్యక్తులతో అధికారస్వరంతో సంభాషించటం(కార్యాలయాలలో)16 పైవారికి మాత్రమే	1	2	3	4	5
interacting with strangers అపరిచితులతో సంభాషించటం	1	2	3	4	5
creating and maintaining family relationships కుటుంబ సంబంధాలు ఏర్పరచుకొని వాటిని కొనసాగించటం	1	2	3	4	5
making and maintaining intimate relationships సన్నిహిత సంబంధాలు ఏరపరచుకొని వాటిని కొనసాగించటం 16 పైవారికి మాత్రమే	1	2	3	4	5
going to school and studying (education) **UNDER 16s ONLY** పాఠశాలకు వెళ్లటం, చదువుకోవటం (విద్య)16 పైవారికి మాత్రమే	1	2	3	4	5
getting and keeping a job (work & employment) **OVER 16s ONLY** ఉద్యోగం సంపాదించుకొని నిలపెట్టుకోవటం(పని మరియు ఉపాధి)16 పైవారికి మాత్రమే	1	2	3	4	5
handling income and payments (economic life) సంపాదనను మరియు చెల్లింపులను నిర్వహించటం (ఆర్థిక విషయాలు)16 పైవారికి	1	2	3	4	5
clubs/organizations (community life) క్లబ్బులు మరియు సంస్థలు (సంఘ జీవనం)16 పైవారికి మాత్రమే	1	2	3	4	5
recreation/leisure (sports/play/crafts/hobbies/arts/culture) వినోదం/విశ్రాంతి (క్రీడలు/ఆటలు/చేతిపనులు/అభిరుచులు/కళలు/సంస్కృతి)	1	2	3	4	5
religious/spiritual activities మతపర/ఆధ్యాత్మిక పనులు 16 పైవారికి మాత్రమే	1	2	3	4	5
political life and citizenship రాజకీయ జీవితం/పౌరసత్వము 16 పైవారికి మాత్రమే	1	2	3	4	5

J. **ENVIRONMENT QUESTIONS: ALL CASES AND CONTROLS** పరిసరాలకు సంభంధించిన ప్రశ్నలు **(అన్ని కేసులు/నియంత్రణలు)**

Being an active, productive member of society includes participating in such things as working, going to school taking care of your home, and being involved with family and friends in social, recreational and civic activities in the community. Many factors can help or improve a person's participation in these activities while other factors can act as barriers and limit participation.

క్రియాశీల, ఉత్పాదక సభ్యుడు కావటం అంటే పని చేయటం, పాఠశాలకు వెళ్లటం, మీ ఇంటి జాగ్రత్తతీసుకోవటం, మరియు కుటుంబం, స్నేహితులతో కలసి సమాజంలోని సాంఘిక, వినోద మరియు పౌర కార్యకలాపాలలో పాల్గొంటు ఉండటంలాంటివి కలిగి ఉంటాయి. ఎన్నో అంశాలు ఈ కార్యకలపాలలో ఒక వ్యక్తిని పాల్గొనేటట్లు సహాయం చేస్తాయి లేదా అభివృద్ధి చేస్తాయి. అయితే ఇతర అంశాలు అవరోధాలుగా పనిచేస్తూ పాల్గొనటాన్ని పరిమితం చేస్తాయి

First, please tell me how often each of the following has been a barrier to your own participation in the activities that matter to you. Think about the past year, and tell me whether each item on the list below has been a problem daily, weekly, monthly, less than monthly, or never. If the item occurs, then answer the question as to how big a problem the item is with regard to your participation in the activities that matter to you.

మొదట, మీకు సంబంధించిన కార్యక్రమాలలో పాల్గొనటానికి క్రిందివాటిలో ఎన్ని మీకు తరచుగా అవరోధాలయ్యాయో నాకు దయచేసి చెప్పండి.గత సంవత్సరం గురించి అలోచించి క్రింద క్రమీకరించిన ప్రతి అంశము ఏ రోజైన, వారంలో , నెలలో, నెలకంటే తక్కువగా అయినా సమస్యగా అయ్యయా లేక అవలేదా అన్నది చెప్పండి. ఒకవేళ ఆ అంశము జరిగివుంటే, మీకు సంబంధించిన కార్యలపానికి ఆ అంశం ఎంత పెద్ద సమస్య అనేది ఈ ప్రశ్నలకు సమాధానమిచ్చి తెలియ చేయండి.

In the past 12 months how often:<br>గత **12** నెలలలో ఎంత తరచుగా :	Daily<br>ప్రతిదినం	Weekly<br>వారానికి	Monthly<br>నెలకు	Less than monthly<br>నెలకుతక్కువ	Never<br>ఎప్పుడూ లేదు	N/A<br>వర్తించదు	When problem occurs, has it been a సమస్య వచ్చినపుడు<br>Big problem<br>పెద్ద సమస్య	 Little Problem<br>చిన్న సమస్య
a. has the availability/accessibility of transportation been a problem for you?<br>రవాణా సదుపాయము/సౌకర్యము కలిగివుండడంలో ఏదైనా సమస్య ఉందా?	1	2	3	4	5	6	1	2
b. has the natural environment – temperature, terrain, climate – made it difficult to do what you want or need to do?<br>మీరు చేద్దామనుకున్న లేదా అవసరమైన పనికి సహజ పర్యావరణంలోని ఉష్ణోగ్రత, ప్రదేశం, వాతావరణం వంటివి మీకు అవరోధంగా ఉండి కష్టం కలిగిస్తోందా?	1	2	3	4	5	6	1	2
c. have other things in your surroundings – lighting, noise, crowds, etc – made it difficult to do what you want or need to do?<br>మీరు చేద్దామనుకున్న లేదా అవసరమైన పనికి వెలుతురు ,శబ్దాలు,జన సమూహాలు వంటి ఇతర విషయాలు మీకు అవరోధంగా ఉండి కష్టం కలిగిస్తున్నాయా?	1	2	3	4	5	6	1	2
d. d. has the information you wanted or needed not been available in a format you can use or understand<br>మీరు కావాలనుకున్న లేదా వాడాలనుకున్న సమాచారము మీరు ఉపయోగించే లేక అర్థం చేసుకొనేందుకు అనువైన స్థితిలో అందుబాటులో ఉందా?	1	2	3	4	5	6	1	2
e. has the availability of health care services and medical care been a problem for you?<br>మీకు ఆరోగ్య రక్షణ సేవలు మరియు వైద్య సంరక్షణ సదుపాయం అందుబాటులో ఏదైనా సమస్య వుందా?	1	2	3	4	5	6	1	2
f Did you need someone else's help in your home and could not get it easily?<br>మీకు మీ ఇంటిలో వేరొకరిది ఎవరిదైనా సహాయం అవసరమా ?మీరు సహాయాన్ని పొందలేకపోతున్నారా?	1	2	3	4	5	6	1	2

g. did you need someone else’s help at school or work and could not get it easily?<br>మీకు పాఠశాలలో లేదా పనిలో వేరొకరిది ఎవరిదైనా సహాయం అవసరమా ?మీరు సహాయాన్ని పొందలేకపోతున్నారా?	1	2	3	4	5	6	1	2
h. Have other people’s attitudes toward you been a problem at home?<br>మీపట్ల ఇంటిలోనిఇతరవ్యక్తుల వైఖరుల వల్ల సమస్యను కలిగివున్నారా?	1	2	3	4	5	6	1	2
i. have other people’s attitudes toward you been a problem at school or work?<br>పాఠశాల లేదా పనిలో మీపట్ల ఇతరవ్యక్తుల వైఖరుల వల్ల సమస్యను కలిగివున్నారా?	1	2	3	4	5	6	1	2
j. did you experience prejudice or discrimination<br>ఎప్పుడైనా పక్షపాతం లేదా వివక్షతను అనుభవించారా?	1	2	3	4	5	6	1	2
k. did the policies and rules of businesses and organizations make problems for you?<br>వ్యాపారము మరియు సంస్థల విధానములు , నిబంధనలు వంటివి మీకు సమస్యలను కలిగించాయా?	1	2	3	4	5	6	1	2
l. did government programs and policies make it difficult to do what you want or need to do?మీరు<br>చేద్దామనుకున్న లేదా అవసరమైన పనికి ప్రభుత్వ కార్యక్రమాలు మరియు విధానాలు మీకు కష్టం కలిగించాయా?	1	2	3	4	5	6	1	2
