# Supplementary material for: Livelihood opportunities amongst adults with and without disabilities in Cameroon and India: A case control study
Source: PLoS One. 2018 Apr 9;13(4):e0194105. doi: 10.1371/journal.pone.0194105 (PMC5890974; doi:10.1371/journal.pone.0194105)
Supplement: S2 File — (PDF) [file pone.0194105.s002.pdf]

ANDRHA PRADESH DISABILITY STUDY 2014
Case Control Questionnaire

COVER SHEET

1 Cluster No. ☐☐ 2 House No. ☐☐ 3 Subject ID No. ☐☐☐

4 Subject Name: ____________________________

5 Interviewer No. ☐☐☐ 6 Date (Day/Month/Year): __ __ / __ __ / __ __

7 Language of Interview:
1 = Telegu
2 = Urdu
3= English

8 Is study subject a case or control?
1 = Case (Person with a disability)
2 = Control

9 What is the study subject's age (years)? ☐☐

10 What is the study subject's gender (observe)
1 = male
2 = female

11 Is the study subject the head of the household 0 = No
1 = Yes

12 Is there another person in this household who has already been interviewed?
0 = No ⟶ **if NO, GO TO Q14**
1 = Yes

13 If yes, what is that person's ID no (check participant card) ☐☐

14 Person interviewed:
1 = Direct interview with case/control
2 = Interview with proxy only — ID of proxy respondant: ☐
3 = Interview with proxy and case/control together — ID of proxy respondant: ☐

A SOCIO-ECONOMIC QUESTIONS

I would first like to check your height and your weight.

1. Interviewer fill in:

1= Weight Measured
2= Weight not measured (wheelchair)
3= Weight not measured (other reason)
Specify ________________________

2 Weight in Kilograms [ ][ ].[ ][ ]

3. Interviewer fill in:

1= Height measured standing
2= Height measured lying down
3= Height not measured
Reason ________________________

4 Height in Centimetres [ ][ ].[ ][ ]

I am now going to ask you a few questions about your household

Note to Interviewer: If there is more than one case or control in the household, this section should be asked to the first person interviewed ONLY

1 How many rooms are there in your household (excluding bathrooms, kitchens, balconies and corridors)? [ ][ ]

2 What is the main source of lighting in your household?

1 = Mains power
2 = Generator/batery/inverter
3 = Kerosene/oil/petrol lamps
4 = Candles
5 = No lighting
6 = Other, specify:
____

3 Does any member of your household own the following (in working order):

	0 = No	1 = Yes
a Radio/HiFi/Stereo	0	1
b TV/VCR/DVD	0	1
c Fridge/Freezer	0	1
d Mobile phone	0	1
e Cupboard	0	1
f Sofa set/armchair	0	1
g Table	0	1
h Motor vehicle incl cars	0	1
i Motorbike	0	1

	0 = No	1 = Yes
j Washing machine	0	1
k Sewing machine	0	1
l Air conditioner	0	1
m Bicycle	0	1
n Stove with gas	0	1
o Stove with electric	0	1
p Computer	0	1
q Kerosene Gas Stove	0	1
r wooden stove	0	1

4 What is the ownership of your household's dwelling?

1= House owned by household
2= Rented house
3= Government owned house
4= Other, specify:
____

B. Water and Sanitation

I would now like to ask you a few qusetions about water and sanitation:
Note to Interviewer: Questions 1,2, 7, 8 and 9 should only be answered once for each household. If there is more than one case or control in the household, this section should be asked to the first person interviewed ONLY

1 What kind of toilet facilities do members of your household usually use
1 = Flush toilet
2 = Traditional latrine
3 =Ventilation improved pit latrine
4 = Bowl/Bucket
5 = Other, Specify: _______________
6 = No toilet → **If NO TOILET GO TO Q7**

2 Do you share this facility with other households?
1 = Used only by your household
2= Shared with other households
3 = Public/ Communal/ Community Latrine

3 Do you use the same toilet facility as other members of your household?
0 = No
1 = Yes → **If YES, go to Q6**

4 Why do you use a different toilet facility from other members of your household (main reason)
1 = It would be physically impossible
2 = I'm not allowed/others would not like it
3 = I might face verbal or physical abuse
4 = I would be embarrased
5 = Other (specify) ____________________

5 What kind of toilet facility do you usually use?
1 = Flush toilet
2 = Traditional latrine
3 =Ventilation improved pit latrine
4 = Bowl/Bucket
6 = No toilet
5 = Other, Specify:

6 Are you usually able to use the toilet facility without you or your clothes coming into contact with feaces
0 = No
1 = Yes

7 What is the main souce of drinking water for members of your household?
1 = Private pipeline
2 = Private well
3 = Public taps/standpipe
4 = Public well
5 = Neighbours
6 = Water vendor
7 = Spring
8 = River/stream/lake
9 = Rainwater
10 = Other, specify:

8 How long does it take to go there, get water and come back?
| | | | minutes

9 Where do members of your household normally bath?
1 = Surface water (eg. Pond, river, sea)
2= Pump or standpipe stored outside compound
3= Piped or stored water inside the house or compound

10 Do you collect water for drinking?
0 = No → **If NO, go to Q15**
1 = Yes

11 Do you collect drinking water from the same source as other members of your household?
0 = No
1 = Yes → **If YES, go to Q17**

12 How long does it take to go there, get water and come back?
| | | | minutes

13 From what source do you usually collect drinking water?
1 = Private pipeline
2= Private hand pump/tap
3 = Private well
4 = Public handpump/tap
5 = Public well
6 = Neighbours
7 = Water vendor
8 = Spring
9 = River/stream/lake
10 = Rainwater
11 = Other, specify:

14	Is this the same source as the water you use for bathing?	0= No<br>1 = Yes
15	Could you collect water from the same source used by other members of your household?	0 = No<br>1 = Yes → **If YES, go to Q17**
16	If no, why not?	1 = It would be physically impossible<br>2 = I'm not allowed/others would not like it<br>3 = I might face verbal or physical abuse<br>4 = I would be embarrased<br>5 = Other (specify)
17	Are you able to access drinking water at home without assistance?	0 = No<br>1 = Yes
18	Have you had diarrhoea (4 or more loose stools within 24 hours) anytime in the past four weeks?	0 = No<br>1 = Yes

C. Marital status, literacy and education (PARTICIPANTS 18 YEARS AND ABOVE ONLY)

Now I would like to ask you a few questions about your living status and education

1 What is your marital status?
- 1 = Married or living together
- 2 = Divorced/seperated
- 3 = Widowed
- 4 = Never married/living together

2 Can you read well, a little or not at all?
- 1 = Well
- 2 = A little
- 3 = Not at all

3 Have you ever attended School
- 0 = No → **If NO, go to Q6**
- 1 = Yes

4 What is the highest level of education you completed
- 1 = Primary
- 2 = Secondary
- 3 = High School
- 4 = University
- 5= Religious School
- 6= No education

5 What was the highest grade that you completed? ☐ → **Go to Q7** If University code 15

6 What is the main reason why you did not receive formal education?
- 1 = absence of school
- 2 = Lack of money
- 3 = Needed to work
- 5 = Education not very useful
- 6 = Being disabled was refused
- 7 = Don't like school
- 8 = Too much household work
- 9 = Family does not allow
- 10 = No transport
- 11 = Other - specify ________________

Ask this question about head of the household (if he/she is NOT the study subject)

7 What is the highest level of education the head of your household completed?
- 1 = Primary
- 2 = Secondary
- 3 = High School
- 4 = University
- 5= Religious School
- 6= No education

8 What was the highest grade that the head of your household completed? ☐

9 Can the head of your household read well, a little or not at all?
- 1 = Well
- 2 = A little
- 3 = Not at all

D. Education questions (CASES/CONTROLS AGED 17 YEARS AND BELOW ONLY)

Note to interviewer: If answered by proxy replace "are you" with "is [name]"

1 Are you currently enrolled in school?
0 = No → **If NO, go to Q7**
1 = Yes

2 Are you enrolled in the same grade as other children your age?
1 = Yes
2= No, lower grade than other children my age
3= No, a higher grade than other children my age

3 Is the school you are in a mainstream/regular school or special school?
1 = Mainstream/regular
2 = Special school
3 = Integrated
4 Mainstream with special/extra classes

4 In the last month of school, how many days did you miss? [ ][ ] days

5 Have you ever repeated a grade at school
0 = No → **If NO, go to Q11**
1 = Yes

6 If yes, how many times have you repeated a grade at school
[ ] → **Go to Q11**

7 If not currently enrolled, have you ever attended school?
0 = No → **If NO, go to Q10**
1 = Yes

8 What is the highest level of education you completed
1 = Primary
2 = Secondary
3 = High School
4 = University
5= Religious School

9 What was the highest grade that you completed? [ ]

10 If you have never attended/are currently not attending school, what is the main reason?
1 = Not enough money
2 = Lack of interest to go to school
3 = Lack of school nearby
4 = Nearby school not accessible
5 = Illness (<1 month)
6 = Illness (> 1 month)
7 =Attendance refused by school
8 = Negative attitudes of other students
9 = Negative attitudes of teachers
10 = Lack of accessible resources to assist child
11 = Child works
12= Other, Specifiy:___________________

Go to Section F

CASES AND CONTROLS (5-17) CURRENTLY ENROLLED IN SCHOOL ONLY

11 If you currently attend school, how often do the following situations happen to you?

	Always	Sometimes	Never	Dont Know
A. If you have a problem at school there are teachers willing to help you	1	2	3	4
B. If you have a problem at school there are friends to help you	1	2	3	4
C. If your friends have a problem at school they come to you for help	1	2	3	4
D. You have friends that you play with at breaktimes	1	2	3	4
E. Your friends look up to you as a leader	1	2	3	4
F. Children hit, hurt or say nasty things to you	1	2	3	4
G. Teachers hit, hurt or say nasty things to you	1	2	3	4
H. You are included in lessons and school activities	1	2	3	4
I. Your school has the right materials to help you learn	1	2	3	4

If CONTROL go to Section F
If CASE continue to next page

CASES (5-17) CURRENTLY ENROLLED IN SCHOOL ONLY

12 I want to know more about your school and whether it is adapted for your needs

	Yes	No	Not Applicable
A. Do you get extra time to complete work or tests	1	2	3
B. Does the teacher teach in a way that makes it easy	1	2	3
C. Do you get extra lessons?	1	2	3
D. Are teaching aids adapted for you eg. Are pictures	1	2	3
E. Does another person in the classroom help you?	1	2	3
F. Is the class environment adapted for you eg.	1	2	3
G. Are communication devices used to help you eg.	1	2	3
H. Is text put in Braille or large font, or audio taped?	1	2	3
I. Are hearing or visual aids used	1	2	3
J. Does someone use sign language with you?	1	2	3
K. Is the playground accessible?	1	2	3
L. Is there an accessible toilet?	1	2	3

13 At school are you able to use the same toilet facility as other pupils?

0 = No
1 = Yes → **If YES, go to Q15**

14 If no, what is the main reason why not?

1 = It would be physically impossible
2= I could not use it without getting myself or my clothes soiled
3 = I'm not allowed/others would not like it
4 = I might face verbal or physical abuse
5 = I would be embarrased
6 = Other (specify) ____________________

15 At school are you able to access drinking water from the same source as other pupils?

0 = No
1 = Yes → **If YES, go to Q17**

16 If no, what is the main reason why not?

1 = It would be physically impossible
2 = I'm not allowed/others would not like it
3 = I might face verbal or physical abuse
4 = I would be embarrased
5 = Other (specify) ______________________

17 At school are you able to wash your hands at the same place as other pupils?

0 = No
1 = Yes → **If YES, GO TO Section F**

18 If no, what is the main reason why not?

1 = It would be physically impossible
2 = I'm not allowed/others would not like it
3 = I might face verbal or physical abuse
4 = I would be embarrased
5 = Other (specify)

GO TO Section F

E Livelihood questions (CASES/CONTROLS AGED 18 YEARS AND ABOVE ONLY)

I would now like to ask you some questions about work

1 Other than domestic work in the household
Have you done any work in the last seven days?
0 = No
1 = Yes → **If YES, Go to Q4**

2 Although you did not work in the last seven days, do you have any job or business from which you were absent for leave, illness, vacation, or any other such reason?
0 = No
1 = Yes → **If YES, Go to Q4**

3 Have you done any work in the last 12 months?
0 = No → **If NO, go to Q8**
1 = Yes

4 What is your occupation, that is, what kind of work do you mainly do?
Code

5 In this work do you :
work on your own/household's business (e.g. Shopkeeper, taxi driver, carpenter, barber) or
work for someone who is not a member of your household (e.g. enterprise, company, government/other individual) or
work on farm owned/rented by yourself or household member

1 = own/household business
2 = non-household member
3 = farm owned/rented by household

6 Do you usually work throughout the year, or do you work seasonally, or only once in a while?
1= Throughout the year
2 = Seasonally/part of the year
3 = Once in a while

7 Are you paid in cash or kind for this work or are you not paid at all?
1 = Cash only
2 = Cash and kind
3 = In kind only
4 = Not paid
Go to Q9

8 If not working, what is the main reason ?
1= Student
2= Childcare/duties/work inside the house
3= Too old / retired
4= Incapable of working, physically
5= Incapable of working, mentally
6= Nobody would give me a job because I am disabled
7= Long illness (>1 month)
8= I am looking for my first job
9= No jobs opportunities in the area
10= Quit/suspended from job
11= Other (please specify): ....

9 Do you receive any of the following benefits

	Yes	No	If yes, from Govt	Non Govt
A. Social security grant	1	0	1	2
B. Disability grant	1	0	1	2
C. Pension	1	0	1	2
D.Family Allowance	1	0	1	2
E. Other (specify)	1	0	1	2

10 AD Are you involved in any of the following

	Yes	No	If yes, run by Govt	Non Govt
A. Self Help Groups	1	0	1	2
B. Microfinance Groups	1	0	1	2
C. Cash for Work schemes	1	0	1	2
D. Other (specify)	1	0	1	2

F. HEALTH AND ANTENATAL CARE

These questions are about your health

F.1. CASES ONLY (all ages)

Note to Interviewer: If participant screened positive via self report say:

Your responses to our earlier questions and examinations indicate that you may have difficulties in certain areas related to your health.

1 What do you think is the cause of the difficulties you face in your health? (tick all that apply)
- 1 = From Birth
- 2 = Trauma
- 3 = Illness
- 4 = Aging
- 5 = Other _____________

2 How old were you when it started? [ ] years
- 00 = from birth
- 99 = Don't know/refused

F2. All CASES AND CONTROLS

1. Have you ever been diagnosed by a medical doctor with any of the following?
(Interviewer: Read out all options and mark all that apply)

	Yes	No
1= High Blood Pessure	1	0
2= Diabetes	1	0
3= Arthritis	1	0
4 =Heart Disease	1	0
5= Asthma, asthmatic bronchitis or allergic bronchitis	1	0
6= Stroke (paralytic attack)	1	0
7= Thyroid problem	1	0
8=Tuberculosis	1	0

	Yes	No
9= COPD	1	0
10= Emphysema	1	0
11= HIV	1	0
12= Cancer<br>If yes, what type of cancer<br>______________________	1	0
13= Any other health condition?<br>If yes, specify ______________________	1	0

2 Have you had any <u>serious</u> health problems that have made you very sick during the last twelve months, including but not limited to those you may have mentioned above?
- 0 = No → **If NO, GO TO SECTION F3**
- 1 = Yes

3 If yes, what type of serious health event(s) or problem(s) did you experience during this period? (tick all that apply)
- 1= Severe Diarrhea (with dehydration or for more than 14 days)
- 2= Acute respiratory tract infection/pneumonia
- 3= Malaria
- 4= Eye Infection/eye problems
- 5= Ear infection/ear or hearing problems
- 6= Malnutrition
- 7= Vaccine-preventable disease (*including measles, chickenpox, mumps, rubella, tetanus, TB, whooping cough)*
- 8= Chronic Illness *(eg. Cancer, HIV*)
- 9= Accident/Injuries
- 10= Jaundice
- 11= Skin Diseases
- 12= Don't know/ no information provided
- 13= Other, specify ________________________

4 Where did you seek advice or treatmer
- 1 = did not seek advice or treatment → **If Response = 1, GO to Q5**
- 2 = village/community health worker or agent
- 3= hospital
- 4= pharmacy
- 5= mobile clinic
- 6= private doctor
- 7= health centre/post (including RMPs)
- 8= traditional healer
- 9= other, specify ....

(2–9) → **All other responses go to Section F3**

5. If you did not seek advice or treatment, what was the reason?
(3 answers possible)

Financial difficulties
1 = I was refused because I had no money (or not enough)
2 = I had difficulty to get food for myself during my stay
3 = I didn't have money to pay for treatment
4 = I didn't have money for medication/objects

Transport, access difficulties
6 = there was no available transportation/it's very far away
7 = I had difficulty to find the money for trasnportation
8= No transport - refused travel on public transport
9 = I had difficulty to find someone to go with me because nobody had time to take me
10 = I did not ask anybody because I felt that it was a waste of time

Difficulties at the health service
11= I did not have the documents required to access health services
12 = there was no available medication
13 = there was no service available for my need (condition)
14 = I was refused because I am disabled
15= attitude of medical staff was negative
16 = The equipment that they gave is not very useful
17 = there is no female professional
18 = Family members did not agree with me seeking treatment
19 = no difficulty
20 =other, specify ____________________________

F3. REPRODUCTIVE HEALTH: MARRIED, SEPARATED, DIVORCED OR WIDOWED FEMALE CASES AND CONTROLS AGED 15 to 49 ONLY. ALL OTHER PARTICIPANTS GO TO SECTION F4

1 Do you have any children? 0 = No → **If NO, go to Q3**
1 = Yes

2 How many children do you have today (excluding those who have died)? [ ]

3 Did you have any pregnancies that ended before term (i.e. Still birth, miscarriage or abortion)? 0 = No → **If NO, GO TO NEXT SECTION F4**
1 = Yes

4 If yes, how many pregnancies ended before term [ ]

F4. PREGNANCY CARE: <u>WOMEN WITH CHILDREN UNDER 5 ONLY. ALL OTHER PARTICIPANTS GO TO SECTION F5</u>

5. Are any of your children aged 5 or younger? 0 = No → **If NO, GO TO NEXT SECTION**
1 = Yes

I would now like to ask you some questions about your children born in the last 5 years. Please answer questions about the last child born in this period

6 Did you see anyone for antenatal care during this time? 0 = No → **If NO, Go to Q7**
1 = Yes

7 Whom did you see?
Anyone else?
Probe to identify each kind of person and record all mentioned

1 = Health personnel/Doctor
2 = Nurse/midwife
3 = Auxiliary Midwife
4 = Traditional Birth Attendant
5 = Community/village health worker
6 = Other (please specify____________)

8 Where did you give birth to [name]?
Probe to identify source. If unable to determine if public/private sector write the name of the place
____

1 = Home (Your home)
2 = Other home
3= Public sector Govt. hospital
4 = Public sector Govt. health centre
5 = Public sector Govt health post
6 = Other public sector (specify____________)
7 = Private medical sector/private hospital clinic
8 = Dispensary
9 = Other private medical sector (specify___________)
10 = Other (specify____________)

9 Who assisted with the delivery of [Name]?
Anyone else?
Probe for the types of person and record all mentioned.
If respondent says no one assisted, probe to determine whether any adults were present at delivery

1 = Doctor
2 = Nurse/Midwife
3 = Auxillary Midwife
4 = Traditional birth attendent
5 = Relative/friend
6 = Other (specify____________)
7 = No one assisted

10 Did [name] ever have any vaccinations to prevent him/her getting diseases, including vaccinations recieved in a national immunization coverage days 0 = No
1 = Yes

G REHABILITATION: CASES ONLY - CONTROLS GO TO SECTION I

	I am now going to ask you some questions about some services specifically for people with disabilities that you may or may not have heard of or have used now or in the past	1.1 Have you ever heard of this type of service?	1.2 Have you ever needed this service?	1.3 Have you ever recieved this service?	1.4 If yes, are you currently recieving or using it?	1.5 If reported needing (Yes to Q1.2) but not receiving a service (No to Q1.3), ask why have you not recieved it?	1.6 If reported once receiving/using service (Yes to Q1.3) but not receiving it now (No to Q1.4), ask why are you no longer recieving it?
		0 = No (go to next service)<br>1 = Yes	0 = No (go to next service)<br>1 = Yes	0 = No → Q1.5<br>1 = Yes → Q1.4	0 = No → Q1.6<br>1 = Yes (go to next service)	1 = Too expensive<br>2 = Too far/no transport<br>3 = Discriminating<br>4 = Communication bariers<br>5 = Don't know where to access<br>6 = Service not available<br>7= Other (specify)______<br>**up to three responses allowed**	1 = Too expensive<br>2 = Too far/no transport<br>3 = Not longer available<br>4 = Communication/language barriers<br>5 = Don't know where to access<br>6 = Not really helping me<br>7 = Not satisfied with services<br>8= No longer need the service<br>9=Broken and unable to repair<br>**up to three responses allowed**
a	**Medical rehabilitation** (e.g. physiotherapy, occupational therapy, speech and hearing therapy etc)	0 1	0 1	0 1	0 1		
b	**Assistive devices service** (e.g. Sign language interpreter, wheelchair, hearing/visual aids, Braille etc.)	0 1	0 1	0 1	0 1		
c	**Specialist educational services (e.g. therapist, school support services)**	0 1	0 1	0 1	0 1		
d	**Vocational Training** (e.g. Employment skills training, etc.)	0 1	0 1	0 1	0 1		
e	**Counselling for person with a disabiliy** (e.g. Physchologist, psychiatrist, counsellor)	0 1	0 1	0 1	0 1		
f	**Counselling for parent/family**	0 1	0 1	0 1	0 1		
g	**Welfare services** (e.g. social worker, disability grant, etc)	0 1	0 1	0 1	0 1		
h	**Health services** (e.g. at a primary health care clinic, hospital, home health care services etc.)	0 1	0 1	0 1	0 1		
i	**Health information** (e.g. From the radio, tv, at schools, clinics, hospital etc.)	0 1	0 1	0 1	0 1		
j	**Traditional healer/faith healer**	0 1	0 1	0 1	0 1		
k	**Legal advice related to having a disability**	0 1	0 1	0 1	0 1		
l	**Specialist health services** (e.g. Surgery, ear/eye medical, psychiatry services)	0 1	0 1	0 1	0 1		

Cluster / House/ID No ___ ___/ ___ ___/ __ __ __

H. ASSISTIVE DEVICES: CASES ONLY - CONTROLS GO TO SECTION I

**Note to Interviewer: Read list of devices that are relevant to difficulty categories of impairment**		1.1 I am going to read you a list of assistive devices. For each please tell me if you use it, need it but don't use it, or don't need it	1.2 If used, is it in good working order?	1.3 If used, where did you get the assitive device?	1.4 If reported, needing but not using:what is the main reason why don't you use it?
Difficulty category	Device	1 = Use it<br>2 = Need it, but don't use it -> Q 1.4<br>3 = Don't need/NA -> next device<br>4 = Don't know what it is -> Next device	1 = Yes<br>0 = No<br>3 = N/A	1 = Private provider<br>2 = Government health service<br>3 = Government service (not health)<br>4 = NGO<br>5 = Friend/relative<br>6 = Other 7 = Don't know<br>(1–7: Next device)	1 = Not really helping me<br>2 = Not satisfied with device<br>3= No longer need the device<br>4=Broken and unable to repair (cost)<br>5=Broken and unable to repair (too far)<br>6= Broken and unable to repair (not available)
Seeing	a. Eye Glasses				
	b. Magnifying glass				
	c. Telescoping Lenses/glasses				
	d. Enlarge print				
	e. Braile				
	f. Other, specify --------------------------				
Hearing	g. Hearing Aid				
	h. Computer				
Mobility	i. Wheel chair				
	j. Crutches				
	k. Walking stick				
	l. White cane				
	m. Guide				
	n. Standing Frame				
	o. Other, specify ----------------------				

2. Do you use any other assitive devicees 0 = No → **If NO, Go to Q4**
1 = Yes → **If YES, Go to Q3**

3. If yes, please tell me what they are: CODE: if other specify _______________
(use device list below)

4. Are there any assistive devices you think you need but do not have? 0 = No → **If NO, go to SECTION I**
1 = Yes → **If YES, Go to Q 5**

5. If yes, please tell me what they are: CODE: if other specify _______________
(use device list below)

DeviceList

Eyeglasses = 1	Communication Board = 8
Hearing Aid = 2	Braille = 9
Wheelchair = 3	Amplified Telephone = 10
Walking Stick = 4	Toilet Seat Raiser = 11
White cane = 5	Bath and shower seats = 12
Crutches = 6	Computers and/or special computer software = 13
Walking Frame = 7	Others (specify) = 14

I. ACTIVITY LIMITATIONS AND PARTICIPATION RESTRICTIONS: ALL CASES AND CONTROLS

1 **ACTIVITY LIMITATION**

I would like to know how difficult it is for you to perform this activity WITHOUT any kind of assistance at all? (Without the use of assistive devices - either technical or personal)

		No difficulty	Moderate difficulty	Severe difficulty	Unable to do	Dont Know
a	watching/looking/seeing	1	2	3	4	5
b	listening/hearing	1	2	3	4	5
c	learning to read/write/count/calculate	1	2	3	4	5
d	acquiring skills (manipulating tools, painting, carving etc.)	1	2	3	4	5
e	thinking/concentrating	1	2	3	4	5
f	reading/writing/counting/calculating	1	2	3	4	5
g	solving problems	1	2	3	4	5
h	understanding others (spoken, written or sign language)	1	2	3	4	5
i	producing messages (spoken, written or sign language)	1	2	3	4	5
j	communicating directly with others	1	2	3	4	5
k	staying in one body position	1	2	3	4	5
l	changing a body position (sitting/standing/bending/lying)	1	2	3	4	5
m	transferring oneself (moving from one surface to another)	1	2	3	4	5
n	lifting/carrying/moving/handling objects	1	2	3	4	5
o	fine hand use (picking up/grasping/manipulating/releasing)	1	2	3	4	5
p	hand & arm use (pulling/pushing/reaching/throwing/catchir	1	2	3	4	5
q	walking	1	2	3	4	5
r	moving around (crawling/climbing/running/jumping)	1	2	3	4	5

PARTICIPATION RESTRICTION

2 Do you have any difficulty performing this activity in your current environment? Now I would like to know whether you have difficulties even with the help of assistive devices or another person

[Current environment where you live, work and play etc for the majority of your time]

		No difficulty	Moderate difficulty	Severe difficulty	Unable to do	Dont Know
a	washing oneself	1	2	3	4	5
b	care of body parts, teeth, nails and hair	1	2	3	4	5
c	toileting	1	2	3	4	5
d	dressing and undressing	1	2	3	4	5
e	eating and drinking	1	2	3	4	5
f	shopping (getting goods and services) **ABOVE 8 ONLY**	1	2	3	4	5
g	preparing meals (cooking) **ABOVE 8 ONLY**	1	2	3	4	5
h	doing housework (washing/cleaning) **ABOVE 8 ONLY**	1	2	3	4	5
i	taking care of personal objects **ABOVE 8 ONLY**	1	2	3	4	5
j	taking care of others	1	2	3	4	5
k	making friends and maintaining friendships	1	2	3	4	5
l	interacting with persons in authority **OVER 16s ONLY**	1	2	3	4	5
m	interacting with strangers	1	2	3	4	5
n	creating and maintaining family relationships	1	2	3	4	5
o	making/maintaining intimate relationships **OVER 16s ONLY**	1	2	3	4	5
p	going to school and studying (education) **UNDER 16s ONLY**	1	2	3	4	5
q	getting and keeping a job (work & employment) **OVER 16s**	1	2	3	4	5
r	handling income and payments **OVER 16s ONLY**	1	2	3	4	5
s	clubs/organisations (community life) **OVER 16s ONLY**	1	2	3	4	5
t	recreation/leisure (sports/play/crafts/hobbies/arts/culture)	1	2	3	4	5
u	religious/spiritual activities **OVER 16s ONLY**	1	2	3	4	5
v	political life and citizenship **OVER 16s ONLY**	1	2	3	4	5

J. ENVIRONMENT QUESTIONS: ALL CASES AND CONTROLS

Being an active, productive member of society includes participating in such things as working, going to school,taking care of your home, and being involved with family and friends in social, recreational and civic activities in the community. Many factors can help or or improve a person's participation in these activities while other factors can act as barriers and limit participation.

First, please tell me how often each of the following has been a barrier to your own participation in the activities that matter to you. Think about the past year, and tell me whether each item on the list below has been a problem daily, weekly, monthly, less than monthly, or never. If the item occurs, then answer the question as to how big a problem the item is with regard to your participation in the activities that matter to you.

**In the past 12 months how often:**	Daily	Weekly	Monthly	Less than monthly	Never	N/A	**When problem occurs, has it been a**	
							Big problem	Little Problem
a. has the availability/accessibility of transportation been a problem for you?	1	2	3	4	5	6	1	2
b. has the natural environment – temperature, terrain, climate – made it difficult to do what you want or need to do?	1	2	3	4	5	6	1	2
c. have other things in your surroundings – lighting, noise, crowds, etc – made it difficult to do what you want or need to do?	1	2	3	4	5	6	1	2
d. has the information you wanted or needed not been available in a format you can use or understand	1	2	3	4	5	6	1	2
e. has the availability of health care services and medical care been a problem for you?	1	2	3	4	5	6	1	2
f. Did you need someone else's help in your home and could not get it easily?	1	2	3	4	5	6	1	2
g. did you need someone else's help at school or work and could not get it easily?	1	2	3	4	5	6	1	2
h. Have other people's attitudes toward you been a problem at home?	1	2	3	4	5	6	1	2
i. have other people's attitudes toward you been a problem at school or work?	1	2	3	4	5	6	1	2
j. did you experience prejudice or discrimination	1	2	3	4	5	6	1	2
k. did the policies and rules of businesses and organizations make problems for you?	1	2	3	4	5	6	1	2
l. did government programs and policies make it difficult to do what you want or need to do?	1	2	3	4	5	6	1	2
